# Short-term disruption of TGF-**β** signaling in adult mice renders the aorta vulnerable to hypertension-induced dissection

**DOI:** 10.1172/jci.insight.182629

**Published:** 2025-02-11

**Authors:** Bo Jiang, Pengwei Ren, Changshun He, Mo Wang, Sae-Il Murtada, María Jesús Ruiz-Rodríguez, Yu Chen, Abhay B. Ramachandra, Guangxin Li, Lingfeng Qin, Roland Assi, Martin A. Schwartz, Jay D. Humphrey, George Tellides

**Affiliations:** 1 Department of Surgery (Cardiac), Yale School of Medicine, New Haven, Connecticut, USA.; 2 Department of Biomedical Engineering, Yale School of Engineering and Applied Science, New Haven, Connecticut, USA.; 3 Program in Vascular Biology and Therapeutics, Yale School of Medicine, New Haven, Connecticut, USA.; 4 Veterans Affairs Connecticut Healthcare System, West Haven, Connecticut, USA.; 5 Department of Medicine (Cardiology),; 6 Department of Cell Biology, and; 7 Yale Cardiovascular Research Center, Yale School of Medicine, New Haven, Connecticut, USA.

**Keywords:** Cell biology, Vascular biology, Cardiovascular disease, Extracellular matrix, Hypertension

## Abstract

Hypertension and transient increases in blood pressure from extreme exertion are risk factors for aortic dissection in patients with age-related vascular degeneration or inherited connective tissue disorders. Yet, a common experimental model of angiotensin II–induced aortopathy in mice appears independent of high blood pressure, as lesions do not occur in response to an alternative vasoconstrictor, norepinephrine, and are not prevented by cotreatment with a vasodilator, hydralazine. We investigated vasoconstrictor administration to adult mice following 1 week of disrupted TGF-β signaling in smooth muscle cells (SMCs). Norepinephrine increased blood pressure and induced aortic dissection by 7 days and even within 30 minutes (as did angiotensin II) that was prevented by hydralazine. Initial medial injury manifested as blood extravasation among SMCs and fibrillar matrix, progressive delamination from accumulation of blood, and stretched or ruptured SMCs with persistent attachments to elastic fibers. Altered regulatory contractile molecule expression was not of pathological importance. Rather, reduced synthesis of extracellular matrix yielded a vulnerable aortic phenotype by decreasing medial collagen, most dynamically basement membrane–associated multiplexin collagen, and impairing cell-matrix adhesion. We conclude that transient and sustained increases in blood pressure can cause dissection in aortas rendered vulnerable by inhibition of TGF-β–driven extracellular matrix production by SMCs.

## Abstract 2

Transient and sustained increases in blood pressure cause dissection in aortas rendered vulnerable by inhibition of TGFβ-driven extracellular matrix production by smooth muscle cells.

## Graphical abstract

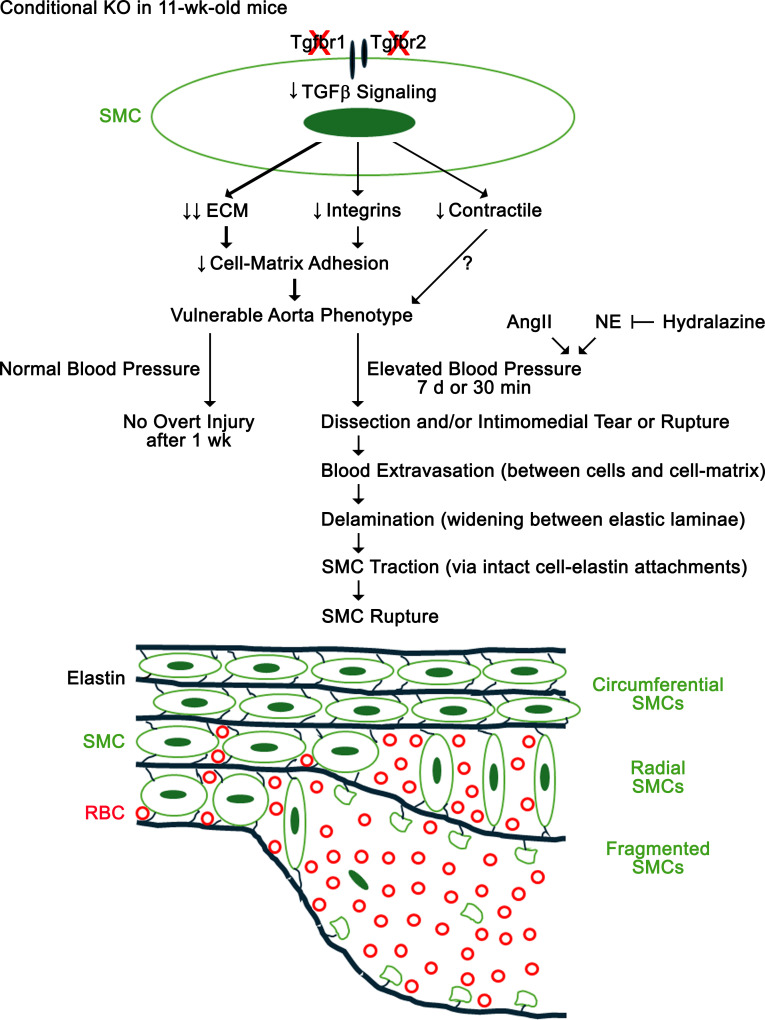

## Introduction

The aorta, as the largest artery in the body subjected to the highest mechanical loads, requires the greatest mechanical strength of any blood vessel. Its complex multilaminar structure can often withstand even supraphysiological loads when constituent cells and extracellular matrix (ECM) exhibit normal organization and function. Rarely, a normal aorta will rupture when acted upon by excessive mechanical stress, as, for example, due to severe blunt trauma. More commonly, structural failure of the aortic wall occurs in response to increased blood pressure within the context of mural defects, typically age-related medial degeneration, local wall thinning secondary to aortic dilatation, or inherited connective tissue disorders. A constellation of life-threatening conditions, including dissection and rupture, termed acute aortic syndrome, results when mechanical stress exceeds wall strength (1). Wall strength is not measured by clinical diagnostic techniques and this lack of knowledge markedly limits our ability to predict complications (2, 3). Thus, the concept of a vulnerable aorta, although challenging to quantify, deserves greater attention.

Experimental models of aortic dissection and rupture have contributed substantially to understanding aorta pathophysiology. A common model is chronic infusion of angiotensin II (AngII) in WT or hyperlipidemic mice because of the simplicity of its design. Infusion of AngII at high rates increases blood pressure and, in a subset of animals, leads to aortic dilatation, dissection, or rupture (4). Yet, aortic lesions do not result after similar pressure elevations induced via infusion of an alternative vasoconstrictor, norepinephrine (NE), and AngII-mediated aortic disease is not prevented by cotreatment with a vasodilator, hydralazine (5, 6). These observations have been interpreted by many in the scientific community as high blood pressure not contributing to aortic dissection and rupture. Given clinical evidence to the contrary, an alternative explanation is that pharmacologically induced hypertension need not injure aortas in the absence of underlying medial vulnerabilities and that, unlike AngII, NE does not induce critical sensitizing mural defects. Indeed, 28-day infusion of NE results in dissection of compromised aortas in *Tgfbr1*-deficient female mice (7), although long-term studies cannot differentiate chronic toxicity via aortic smooth muscle cell (SMC) adrenergic receptors from injury resulting from high blood pressure alone, nor can immediate consequences of medial bleeding be distinguished from effects of subsequent inflammation and vessel wall repair.

We, and others, are interested in roles of TGF-β in aortic development, homeostasis, and disease. Pathogenic variants in genes encoding TGF-β receptors, ligands, and signaling effectors associate with Loeys-Dietz syndrome, a genetic connective tissue disorder predisposing to severe aortopathy (8). Germline deletions in mice of *Tgfbr1* or *Tgfbr2*, encoding TGF-β type I and II receptors, respectively, result in embryonic lethality due to vascular defects, even when restricted to SMC lineages (9, 10). Conditional disruption of TGF-β signaling in SMCs of the developing aorta of 3- to 6-week-old mice results in aortic thickening, dilatation, and dissection (11–13). Yet, little is known about the role of TGF-β in homeostasis of the mature aorta following completion of early postnatal growth, ECM deposition, and acquisition of SMC contractile phenotype.

This study demonstrates that short-term disruption of TGF-β signaling in SMCs of adult mice predisposes to aortic dissection induced by high blood pressure within 7 days of constant infusion and even within 30 minutes of boluses of AngII or NE. Prevention of blood pressure elevation by hydralazine abrogated medial injury. This vulnerable aortic phenotype results from decreased ECM production within the media and impaired cell-matrix adhesion, although persistent cell membrane–elastin attachments likely contribute to traction on and rupture of SMCs. The loss within days of certain collagens, including type XVIII, and certain elastic fiber components, such as microfibrillar-associated protein 4, was unanticipated in view of the far longer half-life for total collagen and negligible elastin turnover in the normal adult aorta.

## Results

### Short-term disruption of TGF-β signaling in SMCs of normotensive adult mice does not induce overt aortopathy.

**Figure 1:**
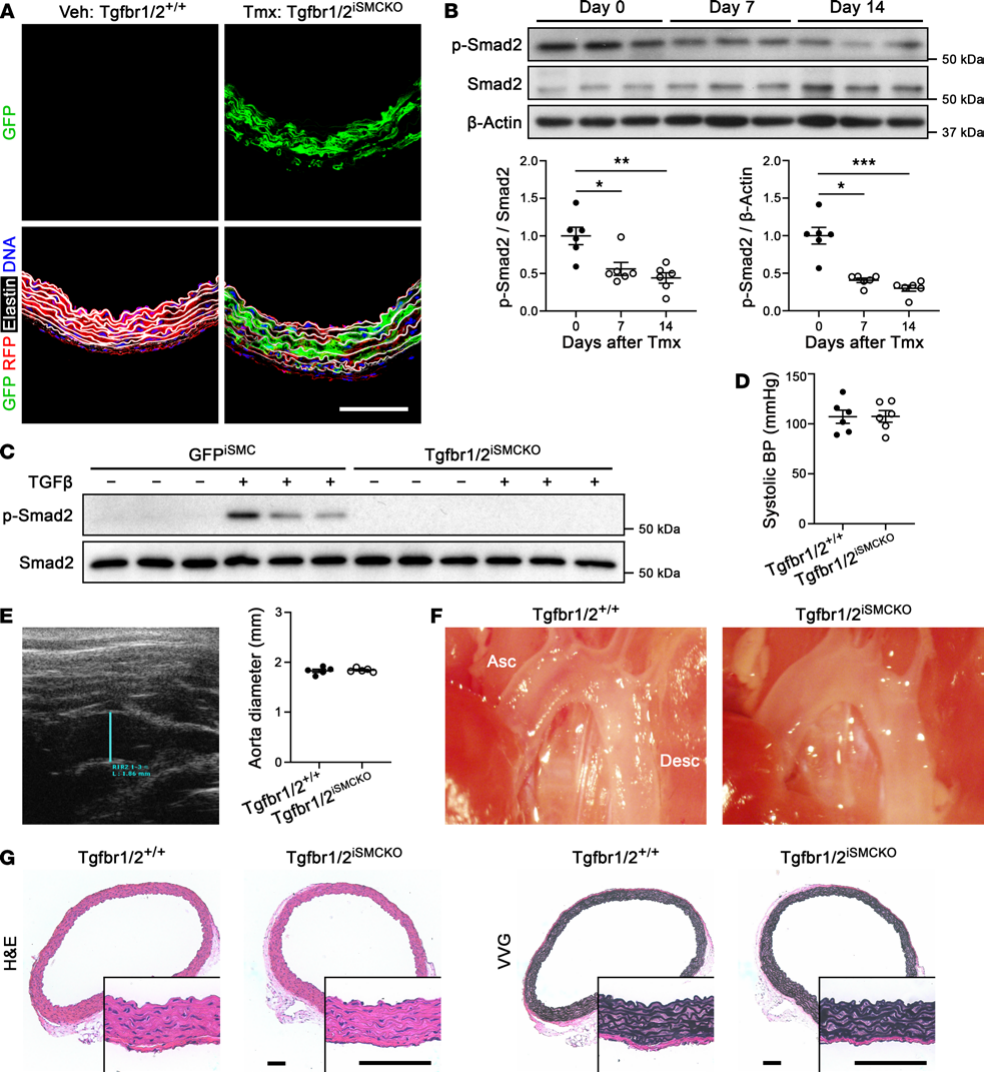
No overt pathology 1 week after disruption of TGF-β signaling in SMCs of mature aortas. Eleven-week-old *Tgfbr1^fl/fl^* *Tgfbr2^fl/fl^* *Myh11*-*CreER^T2^* *mT*/*mG* mice were injected daily with vehicle (Veh, denoted Tgfbr1/2^+/+^) or tamoxifen (Tmx, denoted Tgfbr1/2^iSMCKO^) for 5 days and their ascending aortas were examined at 12 weeks of age. (**A**) Expression of GFP in SMCs with Cre recombination, red fluorescent protein (RFP) in unrecombined cells, Alexa Fluor 633–hydrazide–labeled elastin, and DAPI-labeled nuclei. (**B**) Western blots for indicated proteins in aortas at 0, 7, and 14 days (day 0 denotes untreated) after starting tamoxifen, with densitometry of protein bands relative to loading controls (*n* = 6). (**C**) Similar blots of cultured SMCs isolated from tamoxifen-induced *Myh11*-*CreER^T2^* *mT*/*mG* (denoted GFP^iSMC^) and Tgfbr1/2^iSMCKO^ mice without or with TGF-β exposure at 1 ng/mL for 30 minutes. (**D**) Systolic blood pressure (BP) measured by tail-cuff (*n* = 6). (**E**) Ultrasound examination of ascending aorta diameter (blue line, *n* = 5). (**F**) In situ examination of ascending (Asc) and descending (Desc) thoracic aortas with unremarkable appearances. (**G**) H&E and Verhoeff–Van Gieson (VVG) stains. Scale bars: 100 μm. Data are shown as individual values with mean ± SEM. *P < 0.05, **P < 0.01, ***P < 0.001 by Kruskal-Wallis test with Dunn’s multiple-comparisons test (**B**) or 2-tailed, unpaired Student’s *t* test (**D** and **E**).

We disrupted TGF-β signaling in SMCs of adult mice using a conditional gene deletion strategy that targeted both *Tgbfr1* and *Tgfbr2* to avoid possible aberrant signaling from alternate receptor pairs (14, 15). Compound mutants with Cre recombinase fused to a modified estrogen receptor under control of the *Myh11* promoter enabled SMC specificity (16), while the *mT/mG* reporter allowed identification of cells with transgene recombination by GFP expression (17). Cre activity was induced by tamoxifen administration to 11-week-old *Tgfbr1^fl/fl^* *Tgfbr2^fl/fl^* *Myh11-CreER^T2^* *mT/mG* mice for 5 days, hereafter designated Tgfbr1/2^iSMCKO^. Control littermates were injected with corn oil vehicle (designated Tgfbr1/2^+/+^); other controls with intact TGF-β signaling included C57BL/6J (designated B6 WT) and tamoxifen-induced *Myh11-CreER^T2^* *mT/mG* (designated GFP^iSMC^) mice. Within 7 days of starting tamoxifen (or 2 days after the last dose), 12-week-old Tgfbr1/2^iSMCKO^ mice selectively expressed GFP in most medial cells and demonstrated disrupted TGF-β signaling, as evidenced by decreased phosphorylation of Smad2 (p-Smad2) in aortas in vivo and *Myh11* lineage–marked SMCs in vitro (Figure 1, A–C). Latent and active forms of TGF-β in the medial layer did not change or decreased (Supplemental Figure 1; supplemental material available online with this article; https://doi.org/10.1172/jci.insight.182629DS1). Blood pressure remained unaltered based on tail-cuff measurement, ascending aorta size was unchanged on ultrasound examination, and there was no evident pathology on gross inspection or histological analysis (Figure 1, D–G). In contrast, when TGF-β signaling was disrupted in 4-week-old mice via tamoxifen induction (with similar efficiency of Cre recombination in greater than 90% of SMCs as adult animals), 75% of the Tgfbr1/2^iSMCKO^ mice developed aneurysms, intimomedial tears, and dissection of the thoracic aorta over a 4-week period (Supplemental Figure 2), similar to previous results with deletion of *Tgfbr2* alone (11). Recalling that the aorta matures biomechanically by 8 weeks of age (18), short-term disruption of TGF-β signaling in SMCs of mature aortas does not result in observable aortic disease.

### Continuous infusion of NE induces aortic dissection in adult Tgfbr1/2^iSMCKO^ mice.

**Figure 2:**
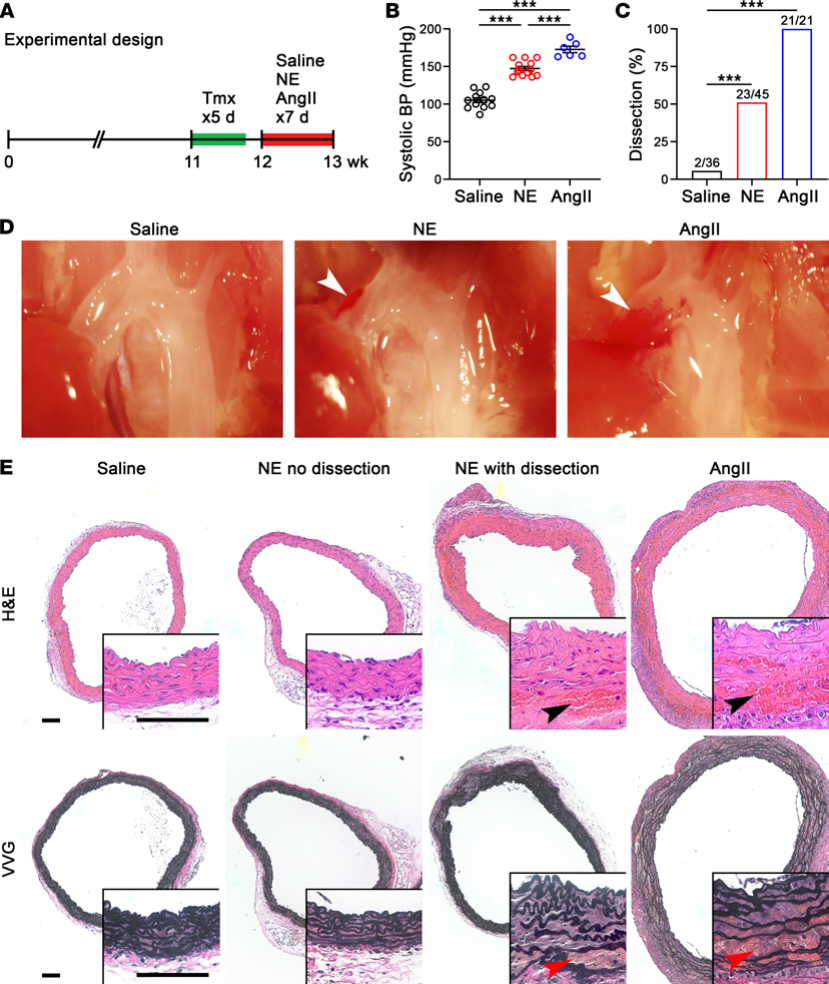
Continuous 1-week infusion of NE induces dissection in vulnerable aortas. (**A**) One week after starting tamoxifen (Tmx), namely 2 days after the last dose, 12-week-old Tgfbr1/2^iSMCKO^ mice were infused with saline, NE at 3.88 μg/kg/min, or AngII at 1 μg/kg/min by osmotic minipump for 7 days and examined at 13 weeks of age. (**B**) Systolic blood pressure (BP) measured by Millar catheter in control and vasoconstrictor-infused animals; saline-treated did not differ from untreated and were combined for greater statistical power (*n* = 6–13). (**C**) Incidence of aortic dissection in saline- (*n* = 2 out of 36), NE- (*n* = 23 out of 45), and AngII-infused (*n* = 21 out of 21) animals. (**D**) In situ examination by dissecting microscope after saline flush via the left ventricle showing mural hematomas of the ascending aorta (arrows). (**E**) H&E and Verhoeff–Van Gieson (VVG) stains confirmed aortic dissections by blood extravasation into the media (arrows) in a subset of NE- and all AngII-infused animals. Scale bars: 100 μm. Data are shown as individual values with mean ± SEM. ***P < 0.001 by 1‑way ANOVA with Tukey’s multiple-comparison test (**B**) or Fisher’s exact test between study groups versus control (**C**).

We investigated whether blood pressure elevation independent of exogenous AngII infusion exposes underlying structural vulnerabilities by infusing 12-week-old Tgfbr1/2^iSMCKO^ mice at a pressor dose of NE known not to induce injury of nonvulnerable aortas (5, 6). A relatively short infusion duration of 7 days was implemented because mild hemorrhagic lesions of the aorta can resolve with few visible sequelae after the longer durations commonly employed in experimental studies (e.g., 28 days) and dissections rarely occur late during this extended period (6, 19). Continuous infusion of NE at 3.88 μg/kg/min for 7 days using an s.c. osmotic minipump increased blood pressure, but to a lesser degree than AngII infusion at the standard dose of 1 μg/kg/min (Figure 2, A and B). Examination under a dissecting microscope after saline flush of luminal blood revealed focal hematomas of the ascending aorta and aortic arch, occasionally extending into the descending thoracic aorta, in 5.6% of saline-, 51.1% of NE-, and 100% of AngII-infused Tgfbr1/2^iSMCKO^ mice (Figure 2, C and D). All 81 saline- and NE-infused animals survived, whereas 3 out of 21 AngII-infused animals died between 3 and 6 days and were confirmed to have ruptured descending thoracic aortas with accumulation of intracavitary blood (Supplemental Figure 3, A and B). Histological analysis verified the hemorrhagic lesions of ascending aortas as dissections, defined as blood extravasation into the media (Figure 2E). Yet, not all intimomedial tears visible by histology associated locally with mural hematomas or medial dissection (Supplemental Figure 3C). We confirmed that NE infusion for 7 days did not result in hemorrhagic lesions in 12-week-old B6 WT mice (Supplemental Figure 3D). In summary, even NE-induced hypertension caused aortopathy in adult Tgfbr1/2^iSMCKO^ mice but not in age- and sex-matched mice with intact TGF-β signaling.

### Bolus injection of NE induces immediate aortic dissection in adult Tgfbr1/2^iSMCKO^ mice.

**Figure 3:**
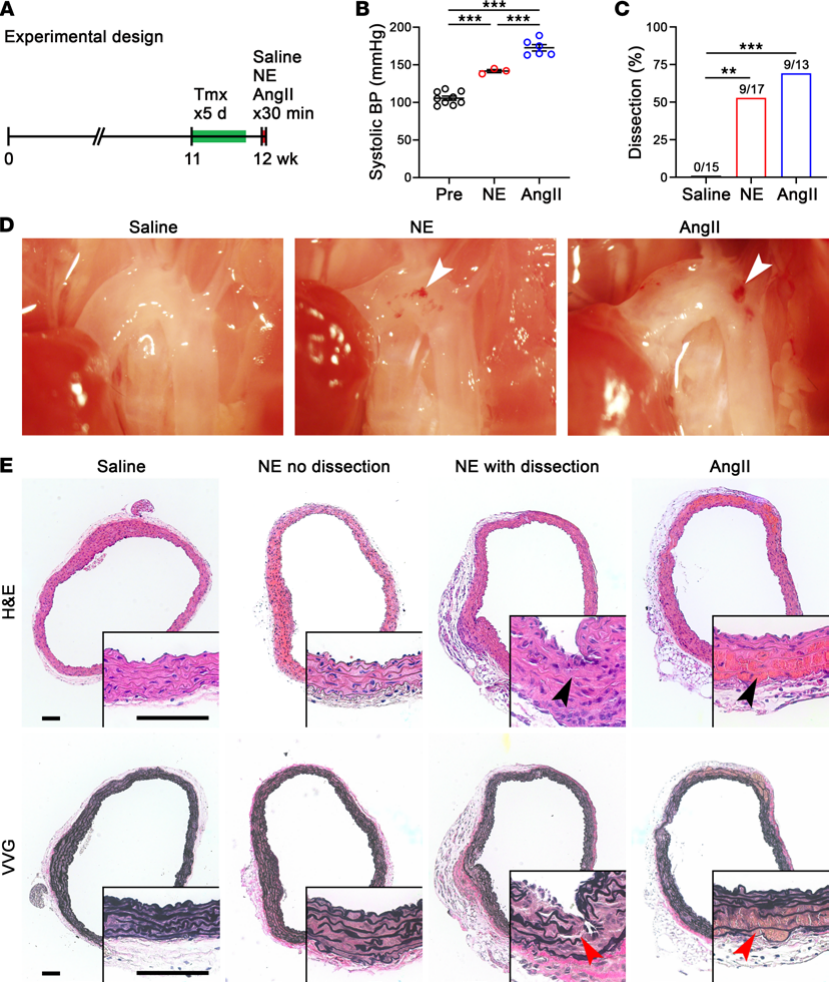
Bolus of NE induces dissection in vulnerable aortas. (**A**) One week after starting tamoxifen (Tmx), or 2 days after the last dose, 12-week-old Tgfbr1/2^iSMCKO^ mice were injected i.p. with saline, NE at 1.28 mg/kg, or AngII at 0.64 mg/kg and examined after 30 minutes. (**B**) Maximum systolic blood pressure (BP) measured by Millar catheter before (Pre) and 30 minutes after injection (*n* = 3–9). (**C**) Incidence of aortic dissection in saline- (*n* = 0 out of 15), NE- (*n* = 9 out of 17), and AngII-injected (*n* = 9 out of 13) animals. (**D**) In situ examination by dissecting microscope after saline flush via the left ventricle showing mural hematomas of the ascending aorta (arrows). (**E**) H&E and Verhoeff–Van Gieson (VVG) stains showing medial dissections (arrows) in a subset of NE- and AngII-injected mice. Scale bars: 100 μm. Data are shown as individual values with mean ± SEM. **P < 0.01, ***P < 0.001 by 1‑way ANOVA with Tukey’s multiple-comparison test (**B**) or Fisher’s exact test between vasoconstrictors versus control (**C**).

To determine whether short-term disruption of TGF-β signaling was sufficient for aortic dissection triggered by elevated blood pressure or whether NE-mediated remodeling of the aorta was also necessary for disease susceptibility, the duration of NE infusion was reduced in steps. In pilot experiments, hematomas occurred in 2 out of 3 aortas at 2 days, 3 out of 4 aortas at 1 day, and 3 out of 4 aortas at 12 hours of NE infusion. We then tested effects of single i.p. injections of NE at a dose of 1.28 mg/kg known to elevate blood pressure acutely in mice (20). Central blood pressure monitoring by invasive catheter via the right carotid artery revealed a rapid increase in blood pressure, peaking within 5 minutes and remaining elevated over 30 minutes, although at lower levels than that induced by single i.p. injection of AngII at a pressor dose of 0.64 mg/kg (20) (Figure 3, A and B, and Supplemental Figure 4, A and B). Examination under a dissecting microscope revealed hematomas of the ascending aorta and/or aortic arch in 0% of saline-, 52.9% of NE-, and 69.2% of AngII-infused Tgfbr1/2^iSMCKO^ mice after 30 minutes (Figure 3, C and D). The hemorrhagic lesions were limited in extent and there were no immediate deaths after boluses of vasoconstrictors. Histology confirmed medial dissection (Figure 3E). Concomitant treatment with a vasodilator, hydralazine at 10 mg/kg i.p., prevented NE-induced pressure elevations and aortic dissections over 30 minutes (Supplemental Figure 4, C–E). Hydralazine did not block blood pressure responses to AngII, however, possibly because of greater vasoconstrictor responses at the doses tested (Supplemental Figure 4F); effects on aortic dissection by this combination of agents were not investigated. Thus, short-term disruption of TGF-β signaling was sufficient to render the aortas vulnerable to dissection induced by high blood pressure independent of possible NE-mediated aortic remodeling.

### Increasing medial delamination associates with traction on and rupture of SMCs.

**Figure 4:**
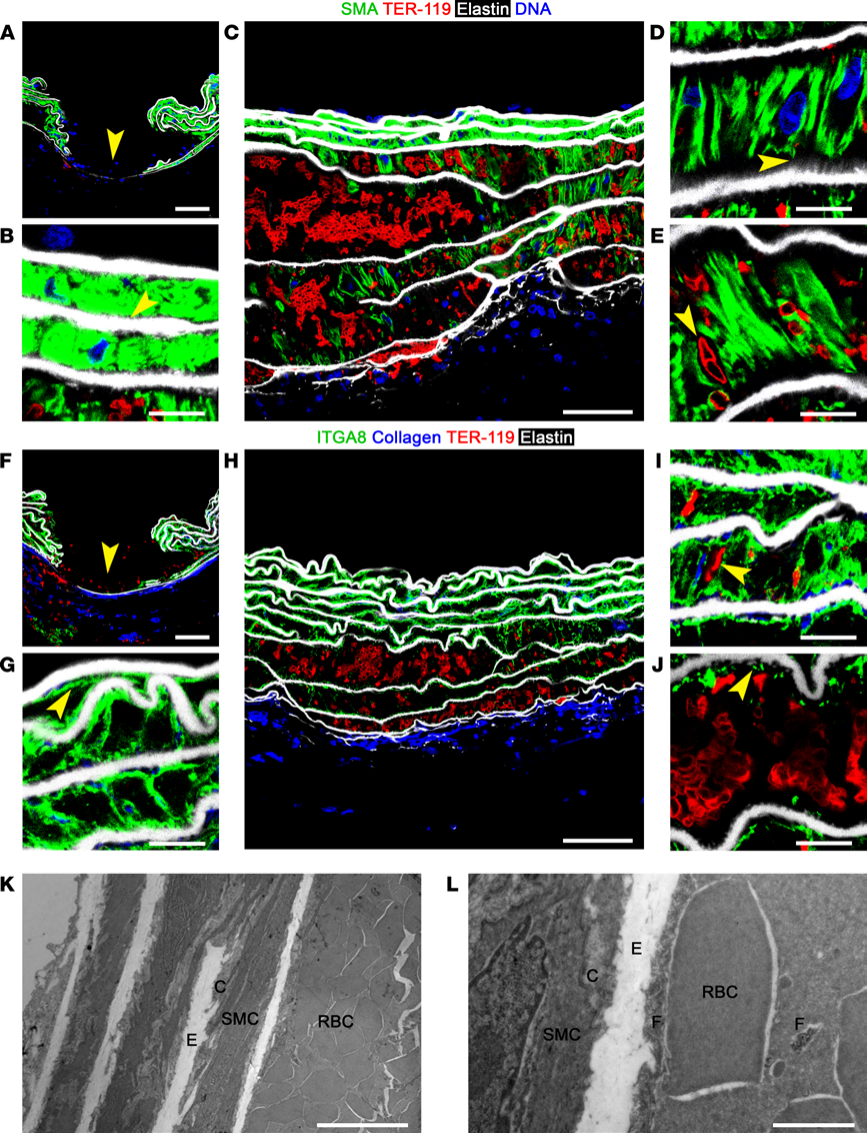
Traction on and rapid fragmentation of SMCs. Twelve-week-old Tgfbr1/2^iSMCKO^ mice were infused with NE at 1.28 mg/kg i.p. for 30 minutes and the ascending aortas examined. Confocal microscopy after labeling smooth muscle α-actin (SMA) for SMC cytoskeleton (green), TER-119 for RBCs (red), Alexa Fluor 633–hydrazide for elastin (white), and DAPI for nuclei (blue) shows (**A**) intimomedial entry tear (arrow), (**B**) nonwidened inner laminae with SMCs adjacent to elastic fibers (arrow), (**C**) varying RBC accumulation in outer laminae, (**D**) widened laminae with radially oriented SMCs attached to ill-defined intralaminar elastic fibers (arrow), and (**E**) RBCs between SMCs (arrow). Alternative labeling of integrin α8 (ITGA8) for SMC plasma membrane (green) and binding of tdTomato-CNA35 to collagen (blue) shows (**F**) intimomedial entry tear (arrow), (**G**) nonwidened laminae with intact SMC plasma membranes (arrow), (**H**) varying RBC accumulation in outer laminae, (**I**) RBCs among SMCs (arrow), and (**J**) widened lamina with attached SMC plasma membrane fragments (arrow) and areas where elastic laminae are stripped clean of cell and fibrillar matrix. Transmission electron microscopy showing (**K**) RBC accumulation in outer laminae and (**L**) nonwidened lamina with SMCs contacting elastic (E) and collagen (C) fibers adjacent to widened lamina with RBCs abutting elastic and collagen fibers and cellular fragments (F). Pressure-fixed (**A**, **F**, **K**, and **L**) and unpressurized (**B**–**E** and **G**-**J**) specimens. Scale bars: 50 μm (**A**, **C**, **F**, and **H**), 10 μm (**B**, **D**, **E**, **G**, and **I**–**K**), and 2 μm (**L**).

Dissected aortas after vasoconstrictor infusion for 30 minutes were examined further to define immediate histopathological outcomes prior to time-dependent responses to injury, such as apoptosis, inflammation, and fibrosis. Specimens were perfusion-fixed at physiological pressure to straighten the medial laminae (Supplemental Figure 5); because this process could cause distension artifacts, we examined both pressure-fixed and unpressurized specimens. Partial tears through the intima and subjacent media marked by thrombus served as entry sites for blood extravasation into the media, but an intact external elastic lamina and adventitia prevented free rupture (Supplemental Figure 6, A and B). Confocal studies with reagents specific for SMC cytoskeleton, RBCs, and elastin revealed disease heterogeneity, with blood tracking circumferentially and axially from intimomedial entry tears along the outer laminae while sparing inner laminae (Figure 4, A–E). Nonwidened laminae consisted of tightly packed SMCs bordered by elastic fibers. In moderately widened laminae there was separation of neighboring SMCs from each other with intervening RBCs; the long axis of SMCs changed from circumferential to radial with persistent attachment to ill-defined extensions of elastic laminae (intralaminar elastic fibers). In contrast, markedly widened laminae were filled with RBCs amid infrequent SMC fragments. Similar heterogeneous effects were found with alternative reagents labeling SMC plasma membrane and collagen; these components lined elastic fibers in areas without dissection, while individual or small groups of RBCs infiltrated between SMCs and fibrillar matrix in laminae with mild dissection and SMC membrane fragments adhered to elastic fibers in regions with marked delamination (Figure 4, F–J). Transmission electron microscopy confirmed displacement of SMCs by RBCs with residual cell and matrix fragments attached to widened laminae but unremarkable relationships of SMCs with elastic and collagen fibers in adjacent non-widened laminae (Figure 4, K and L). In contrast with hemorrhagic aortic lesions, circumferentially oriented SMCs were uniformly attached to ECM and neighboring cells in aortas prior to NE administration or without dissection despite pharmacologically induced pressure elevation (Supplemental Figure 6, C and D). Combining a series of images at different focal planes (*Z*-stack) enhanced visualization of progressive medial delamination by intralaminar accumulation of blood, radial reorientation and rupture of SMCs, and persistent attachment of cells and cell fragments to elastic fibers (Supplemental Figure 7). These findings suggest underlying TGF-β–dependent defects in cell-cell or cell-matrix adhesion as well as contractility or cytoskeletal strength, respectively.

### Short-term disruption of TGF-β signaling does not impair bulk mechanical properties.

**Figure 5:**
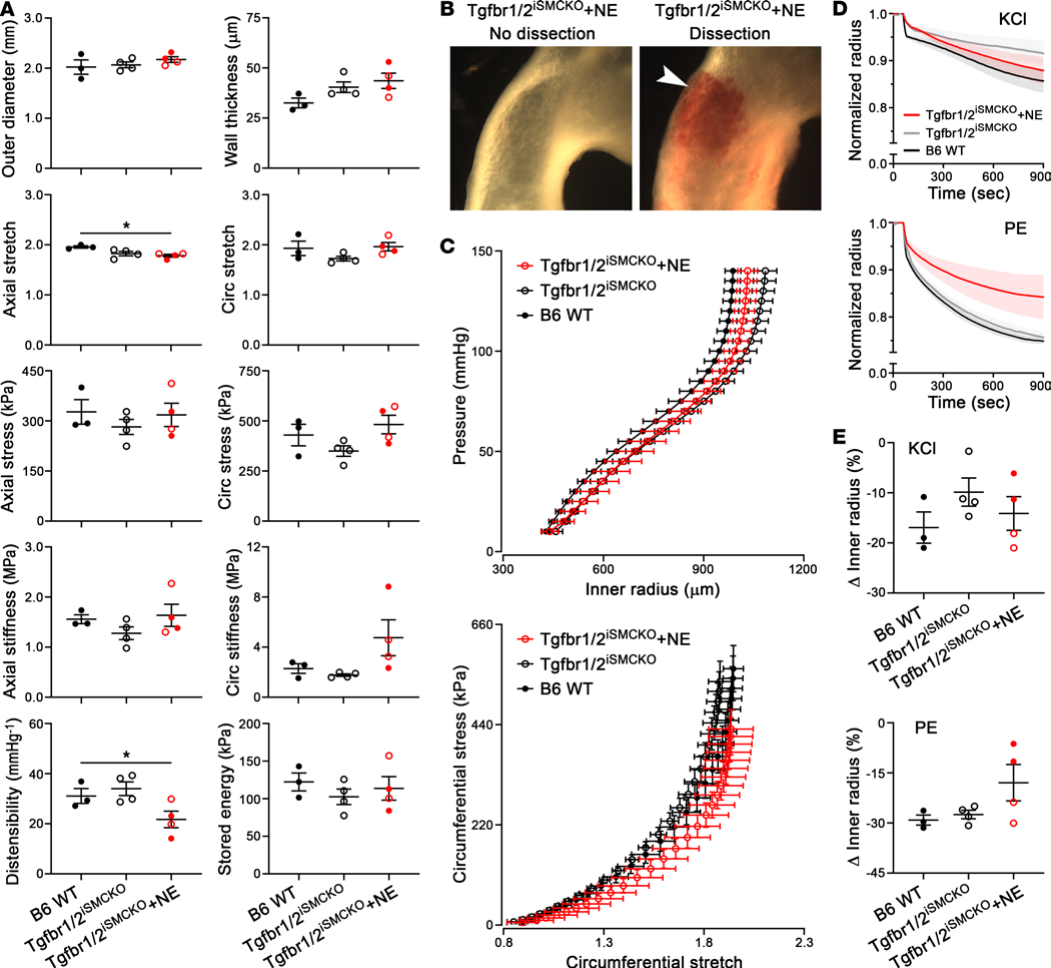
Limited impairment of bulk biomechanical properties 1 week after disrupting TGF-β signaling in mature aortas. Vessel-level biomechanical testing was performed on ascending aortas from untreated B6 WT mice and noninfused or 7-day NE–infused Tgfbr1/2^iSMCKO^ mice. There were 2 classes of tests performed: “active” and “passive.” During active tests, each vessel was held at its individual axial stretch and common luminal pressure of 90 mmHg when exposed to vasoactive agonists. During passive tests, vessels were cyclically pressurized 10–140 mmHg while held at 1 of 3 specimen-specific axial stretches, and then cyclically stretched axially while held at 1 of 4 different common pressures. Data from these 7 protocols were used to calculate material parameters describing the wall mechanics for each vessel. (**A**) Outer diameter, wall thickness, axial and circumferential (circ) stretch, mean wall stress, and material stiffness, distensibility, and stored energy at group-specific systolic pressures (107 mmHg for B6 WT, 108 mmHg for Tgfbr1/2^iSMCKO^, and 147 mmHg for Tgfbr1/2^iSMCKO^ + NE). (**B**) Unpressurized ascending aortas from NE-infused Tgfbr1/2^iSMCKO^ mice without or with dissection (arrow). (**C**) Overlapping pressure-radius and circumferential stress-stretch curves among the 3 groups. (**D**) Vasoconstriction, against a fixed pressure at the in vivo axial stretch, responses to KCl and phenylephrine (PE) assessed by reduction of normalized inner radius over time. (**E**) Steady-state change in inner radius in response to KCl and PE. Data are shown as individual values with mean ± SEM (**A** and **E**) or mean ± SEM with connecting lines (**C** and **D**). *n* = 3–4 per group, NE infusion resulted in no dissection (open red symbols, *n* = 2) or dissection (filled red symbols, *n* = 2). *P < 0.05 by 1‑way ANOVA with Tukey’s multiple-comparison test (**A** and **E**) or Kruskal-Wallis test with Dunn’s multiple-comparison test (**A**: wall thickness and axial stiffness).

We investigated whether vulnerability of the aortic wall following TGF-β disruption and 7 days of NE infusion related to changes in bulk structural or material properties. Passive testing (i.e., without sustained SMC contractility) of ascending aortas was performed ex vivo using a computer-controlled multiaxial device (21). Vessel segments did not leak when extended axially and pressurized over physiological ranges, implying overall structural integrity. Most biomechanical metrics remained similar despite the presence of small dissections in a subset of aortas from NE-infused animals (Figure 5, A and B). A modest decrease in axial stretch and distensibility of aortas from NE-infused Tgfbr1/2^iSMCKO^ mice suggested modest elastic fiber breaks or accumulation of nonelastic material such as glycosaminoglycans, blood, or debris. Elastic energy storage, a key function of the aortic wall stemming primarily from intact elastic laminae, was yet unchanged. The minimal differences in mechanical properties were also illustrated by overlapping pressure-radius (structural behavior) and stress-stretch (material behavior) curves (Figure 5C). Active testing (i.e., during induced SMC contractility) revealed decreased ex vivo responses to phenylephrine in ascending aortas of NE-infused Tgfbr1/2^iSMCKO^ mice, suggesting desensitization to adrenergic stimuli rather than global impairment in contractile function, as KCl responses were not affected (Figure 5, D and E). Although biomechanical properties averaged over an entire vessel segment showed only small differences after short-term disruption of TGF-β signaling, this does not exclude local microdefects with a potential to initiate and propagate aortic dissection in response to a hypertensive stimulus.

### Disrupted TGF-β signaling alters transcription of ECM and contractile molecules.

**Figure 6:**
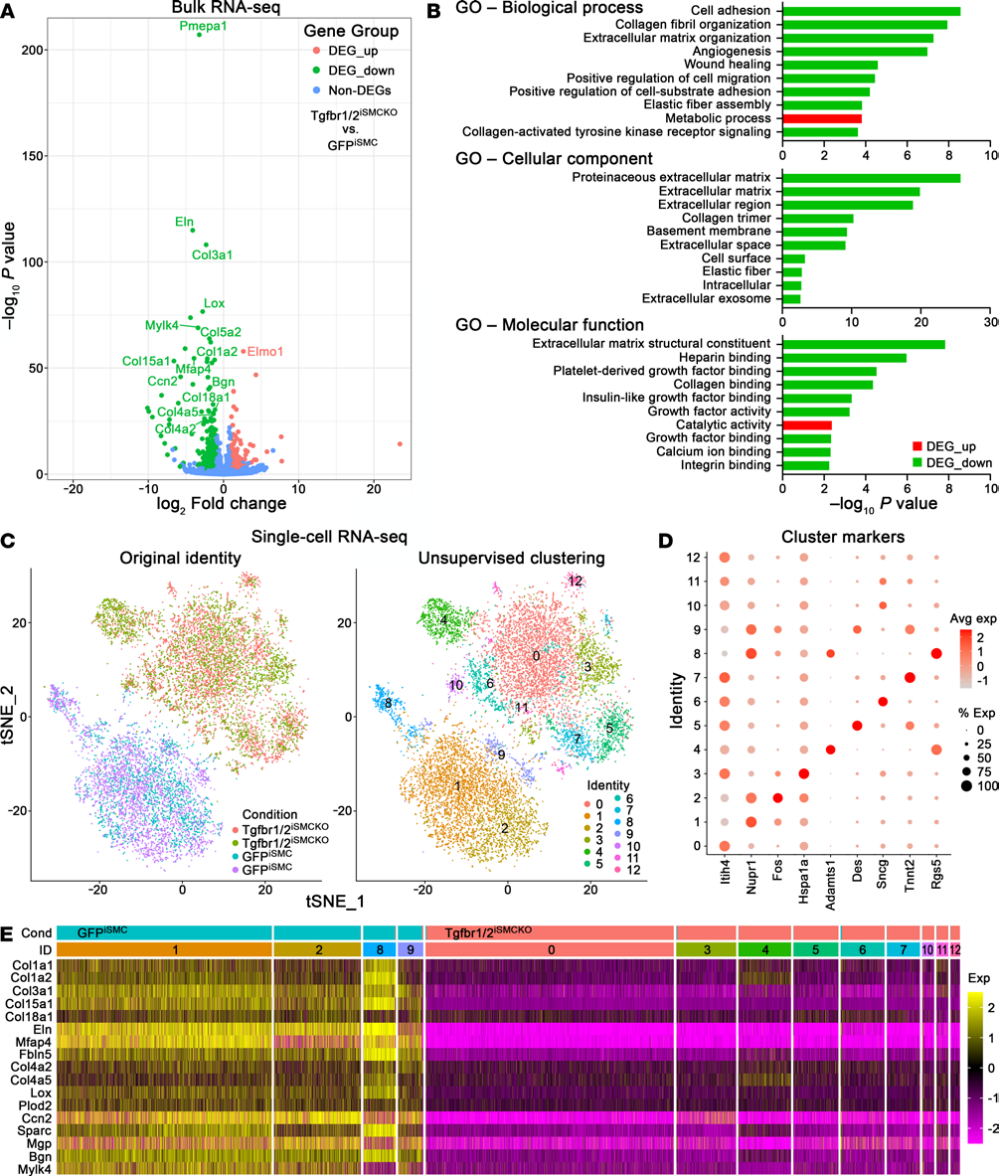
Whole transcriptome profiling 1 week after disrupting TGF-β signaling in SMCs of mature aortas. *Myh11* lineage–marked SMCs were isolated from thoracic aortas of 12-week-old GFP^iSMC^ and Tgfbr1/2^iSMCKO^ mice. Bulk RNA-seq (*n* = 12) shown as (**A**) volcano plot of differentially expressed genes (DEGs) and (**B**) gene ontology (GO) enrichment analysis. Single-cell RNA-seq (*n* = 4) shown as (**C**) *t*-distributed stochastic neighbor embedding (tSNE) plots with 13 clusters identified and cells distinctly partitioned by experimental condition, (**D**) dot plot of log_2_ average expression and percentage expression of cluster markers (markers for 4 clusters containing <3% of total cells are not shown), and (**E**) heatmap of selected ECM and regulatory contractile molecules (identified as differentially expressed in bulk RNA-seq analysis) illustrating uniformly decreased expression in almost all cells with TGF-β signaling disruption irrespective of SMC subclusters (clusters 0, 3–7, and 10–12).

To identify transcriptional responses associated with short-term disruption of TGF-β signaling and vulnerability to hemodynamic stresses, we performed whole transcriptome profiling. Bulk RNA sequencing (RNA-seq) of thoracic aortas from 12-week-old Tgfbr1/2^iSMCKO^ versus B6 WT mice revealed 840 differentially expressed genes with greater than 2-fold change, including an ECM (*Col15a1*) and a regulatory contractile (*Mylk4*) transcript as the most significantly downregulated (Supplemental Figure 8A). Gene ontology profiling identified cell adhesion and ECM among overrepresented terms (Supplemental Figure 8B). Since analysis of whole aortic tissue may be confounded by transcript expression from nonrecombined SMCs and cell types other than SMCs, we analyzed isolated cells after cryophilic enzyme digestion at 4°C to prevent cell stress artifacts (22). Bulk RNA-seq of *Myh11* lineage–marked SMCs from 12-week-old Tgfbr1/2^iSMCKO^ versus GFP^iSMC^ mice showed 597 differentially expressed genes, of which most were downregulated, including those for diverse ECM components — fibrillar (e.g., *Col1a2*, *Col3a1*) and nonfibrillar (e.g., *Col15a1*, *Col18a1*) collagens, elastic fibers and microfibrils (e.g., *Eln*, *Mfap4*), basement membrane (e.g., *Col4a2*, *Col4a5*), enzymes for ECM organization (e.g., *Lox*), matricellular molecules (e.g., *Ccn2*), and various proteoglycans (e.g., *Bgn*) — as well as regulatory contractile molecules (e.g., *Mylk4*) (Figure 6A). Gene ontology enrichment analysis again identified ECM and cell adhesion themes predominating among overrepresented terms (Figure 6B). Changes in gene expression were similar in SMCs from ascending/arch and descending thoracic segments (Supplemental Figure 8C). The differential expression of genes in isolated SMCs only partially overlapped with that of aortas, however, because expression of many ECM molecules (but not *Col15a1* and *Col18a1*) predominated in fibroblasts and thus confounded whole tissue analysis (Supplemental Figure 8, D–F). Single-cell RNA-seq analysis of *Myh11* lineage–marked SMCs from ascending/arch segments revealed a primary partition of populations by TGF-β signaling state and further clustering of cells within each experimental condition (Figure 6, C and D). Gene expression of selected ECM and regulatory contractile molecules, quantified as differentially expressed by bulk RNA-seq, also showed clear separation by experimental condition in single-cell RNA-seq analysis, demonstrating ubiquitous effects of TGF-β signaling disruption among SMC subpopulations (Figure 6E). The predominant loss of transcripts in SMCs from Tgfbr1/2^iSMCKO^ versus GFP^iSMC^ mice was unlikely due to dying cells, as cell viability and RNA quality control metrics were similar between experimental groups and replicates (Supplemental Figure 9). Together, these unbiased analyses document rapid downregulation of genes for many ECM molecules secreted by SMCs, likely affecting cell-matrix interactions with relatively few changes in contractile molecules.

### Altered expression of regulatory contractile molecules is not of pathological importance.

**Figure 7:**
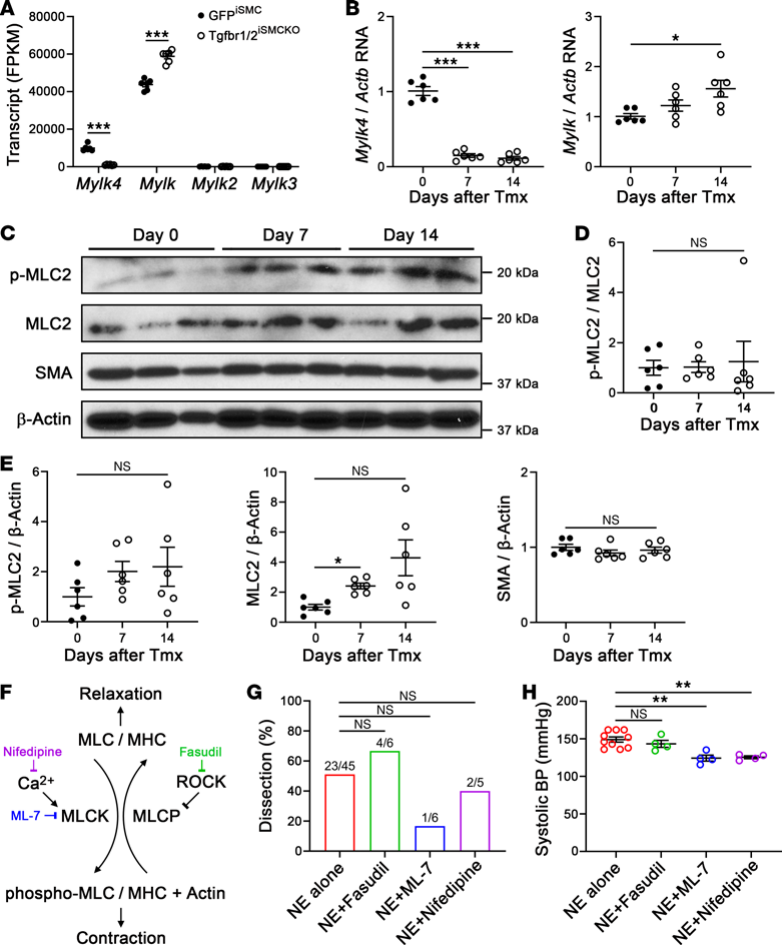
Altered expression of regulatory contractile molecules does not substantially contribute to disease phenotype. Thoracic aortas were analyzed after various imposed conditions. (**A**) Bulk RNA-seq for *Mylk4*, *Mylk*, *Mylk2*, and *Mylk3* as fragments per kilobase million (FPKM) in GFP^+^ SMCs from GFP^iSMC^ and Tgfbr1/2^iSMCKO^ mice (*n* = 6). (**B**) Quantitative RT-PCR for *Mylk4* and *Mylk*, relative to *Actb*, in aortas of Tgfbr1/2^iSMCKO^ mice at 0–14 days (day 0 denotes untreated) after tamoxifen (Tmx, *n* = 6). (**C**) Western blots for phospho-myosin light chain-2 (p-MLC2), MLC2, smooth muscle α-actin (SMA), and β-actin in aortas of Tgfbr1/2^iSMCKO^ mice at 0–14 days after tamoxifen. Densitometry relative to (**D**) MLC2 or (**E**) β-actin (*n* = 6). (**F**) Phosphorylation of MLC by myosin light chain kinase (MLCK) leads to myosin heavy chain (MHC)-actin–mediated contraction, whereas dephosphorylation by myosin light chain phosphatase (MLCP) enables relaxation. Vasoconstrictors activate MLCK via Ca^2+^ or inhibit MLCP via Rho-Rho kinase (ROCK); p-MLC and SMC contractility are inhibited by the MLCK inhibitor, ML7, the Ca^2+^ channel blocker, nifedipine, and the ROCK inhibitor, fasudil. (**G**) Incidence of aortic dissection in Tgfbr1/2^iSMCKO^ mice infused with NE alone (*n* = 23 out of 45) or with concomitant treatment with fasudil (*n* = 4 out of 6), ML-7 (*n* = 1 out of 6), or nifedipine (*n* = 2 out of 5) for 7 days. (**H**) Systolic blood pressure (BP) measured by tail-cuff after NE infusion with and without pharmacological agents for 7 days (*n* = 4–10). Data are shown as individual values with mean ± SEM. *P < 0.05; **P < 0.01; ***P < 0.001; or not significant (NS) by 2‑way ANOVA with Šidák’s multiple-comparison test (**A**), 1‑way ANOVA with Tukey’s multiple-comparison test (**B** [left panel], **E** and **H**), Kruskal-Wallis test with Dunn’s multiple-comparison test (**B** [right panel], **D**), or Fisher’s exact test between combined treatments vs. NE alone (**G**).

We initially considered whether changes in SMC contractile or synthetic phenotypes contributed to aortic vulnerability, as transcripts involved in these functions were among the top differentially expressed genes by RNA-seq of aortas and SMCs. We examined the role of *Mylk4*, which encodes myosin light chain kinase-4, because maximal SMC contraction prevents medial delamination ex vivo (23). Quantitative RT-PCR analysis of whole aortic tissue confirmed decreased *Mylk4*, although *Mylk* encoding smooth muscle myosin light chain kinase, the dominant isoform of the enzyme in SMCs, was increased, corresponding to bulk RNA-seq analysis (Figure 7, A and B). As expected, *Mylk2* and *Mylk3*, encoding isoforms expressed in skeletal and cardiac muscle, respectively, were not detected in SMCs. Western blot analysis showed persistent activation of myosin light chain-2 (the target of myosin light chain kinases), suggesting that downregulation of *Mylk4* did not have functional impact (Figure 7, C–E). We further tested whether inhibition of SMC contraction potentiated effects of TGF-β signaling disruption using various pharmacological agents as a gain-of-function (more aptly gain-of-dysfunction) approach (Figure 7F). Inhibition of myosin light chain kinase by ML-7 or indirectly by a calcium channel blocker, nifedipine, or a Rho kinase inhibitor, fasudil, did not significantly alter the incidence of NE-triggered aortic dissection, although with a confounding lowering of blood pressure by the former 2 agents (Figure 7, G and H). The limited data with at least 1 myosin light chain modulator in the context of persistently increased blood pressure from NE suggest that changes in SMC contractility do not play a crucial role in the vulnerable aorta phenotype of this model.

### Short-term TGF-β signaling disruption in SMCs of mature aortas decreases medial ECM.

**Figure 8:**
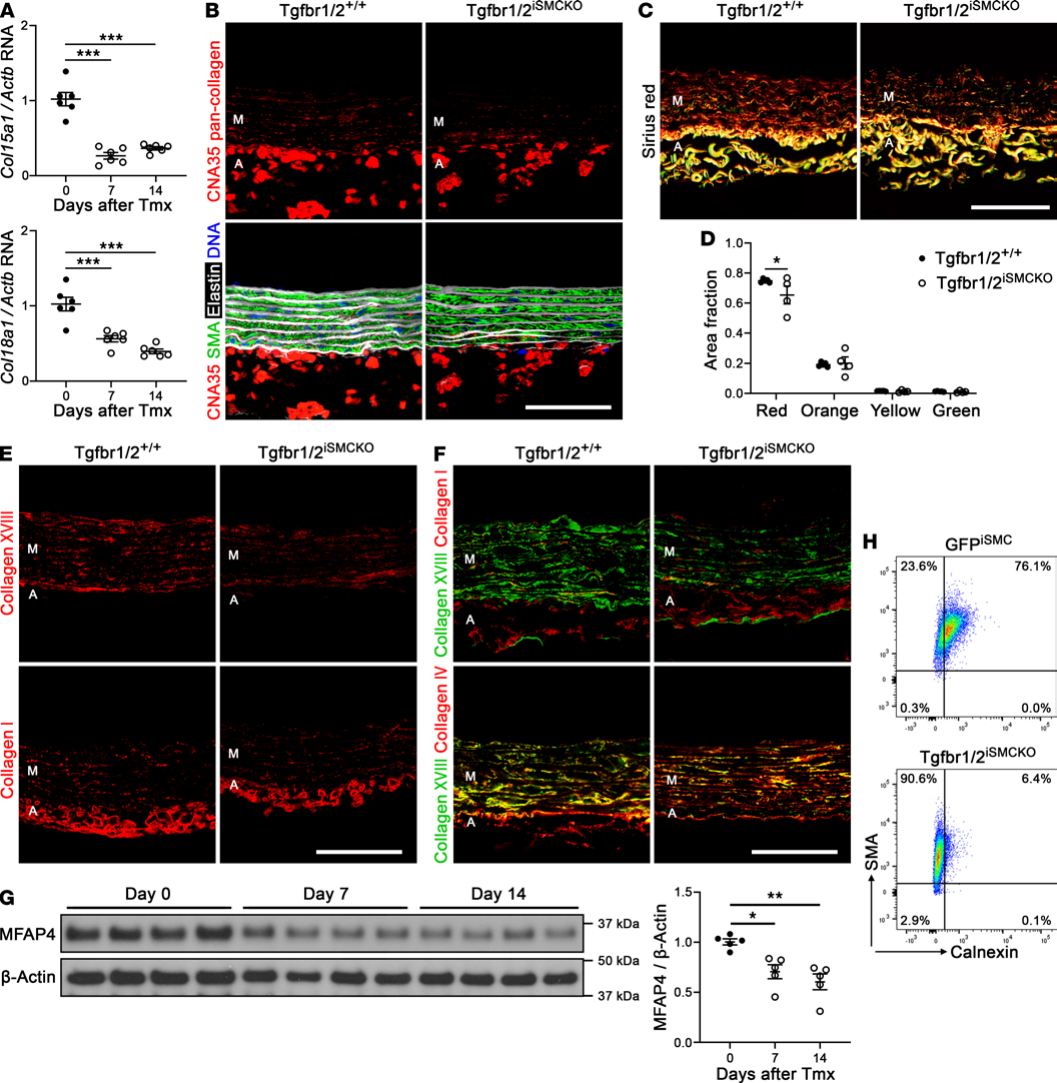
Decreased medial collagen 1 week after disrupting TGF-β signaling in SMCs of mature aortas. Thoracic aortas were analyzed after various imposed conditions. (**A**) Quantitative RT-PCR for *Col15a1* and *Col18a1* transcripts, relative to *Actb*, at 0–14 days (day 0 denotes untreated) after starting tamoxifen (Tmx, *n* = 6). Collagen protein studies were performed in 12-week-old Tgfbr1/2^+/+^ and Tgfbr1/2^iSMCKO^ mice at 7 days after starting tamoxifen. (**B**) Confocal microscopy after labeling with the collagen-binding probe tdTomato-CNA35 (red) alone or overlaid with smooth muscle αactin (SMA) for SMCs (green), Alexa Fluor 633–hydrazide for elastin (white), and DAPI for nuclei (blue). (**C**) Picrosirius red staining for collagen under polarized light. (**D**) Quantification of Picrosirius red polarized colors as fraction of media area (*n* = 4–5). (**E**) Presence of collagen XVIII (red) or collagen I (red). (**F**) Overlay of collagen XVIII (green) and I (red) or collagen XVIII (green) and IV (red) demonstrating colocalization (yellow). Images are from formalin-fixed, paraffin-embedded sections (**B**, **C**, and **E**) and frozen, OCT-embedded sections (**F**) of pressure-fixed (**B**, **E**, and **F**) and unpressurized (**C**) specimens. The media (M) and adventitia (A) are identified by the presence or absence of elastic laminae. Scale bars: 50 μm. (**G**) Western blots for microfibrillar-associated protein 4 (MFAP4) and β-actin in media at 0, 7, and 14 days after starting tamoxifen, with densitometry of protein bands relative to loading controls (*n* = 4). (**H**) Intracellular expression of the contractile filament marker SMA versus the endoplasmic reticulum marker calnexin by flow cytometry consistent with reduction in synthetic state of SMCs from Tgfbr1/2^iSMCKO^ vs. GFP^iSMC^ mice. Data are shown as individual values with mean ± SEM. *P < 0.05; *P < 0.01; ***P < 0.001 by 1‑way ANOVA with Tukey’s multiple-comparison test (**A** and **G**) or 2‑way ANOVA with Šidák’s multiple-comparison test (**D**).

Based on the whole transcriptome findings, we further investigated the expression of ECM molecules synthesized by SMCs. Quantitative RT-PCR revealed a rapid decline in *Col15a1* and *Col18a1* in thoracic aortas within 7 days of TGF-β receptor deletion (relevant for 30-minute vasoconstrictor experiments) and similarly at 14 days (relevant for 7-day vasoconstrictor experiments) (Figure 8A). Other collagen transcripts were not measured because of the confounding influence of the adventitia. Although the greater collagen content of the adventitia overshadows that in other vascular wall compartments, medial collagen protein is detected after overexposure of microscopy images (24). Medial collagen appeared decreased by confocal microscopy using a pan collagen–binding probe and was confirmed as modestly reduced by quantitative analysis of histological stains (Figure 8, B–D). Assessment of individual collagens for which suitable reagents were available showed varying results, including substantially less collagen XVIII but less reduction in collagen I and III (Figure 8E and Supplemental Figure 10, A and B). In keeping with SMC specificity of *Col18a1* by single-cell RNA-seq analysis, collagen XVIII was not detected in the adventitia except in covering mesothelial cells (visceral pericardium). Collagen XVIII colocalized with collagen IV, but not collagen I, suggesting interactions with SMC basement membrane (Figure 8F). Similar colocalization was seen with another basement membrane component, perlecan (Supplemental Figure 10, C and D). Collagen IV and perlecan encircled SMCs but did not appear decreased within 7 days of TGF-β receptor deletion (Supplemental Figure 10, E and F). Although collagens and elastin were not reliably extracted from aortic media to verify expression changes by Western blot, satisfactory bands for microfibrillar-associated protein 4 demonstrated a rapid decrease in levels (Figure 8G). Decreased synthesis of ECM molecules associated with phenotypic modulation of SMCs to a less synthetic state, as evidenced by a decreased proportion of endoplasmic reticulum relative to contractile filament markers (Figure 8H). Thus, various medial ECM proteins decreased within 7 days of disrupting TGF-β signaling in SMCs under physiological hemodynamic conditions.

### Inhibition of ECM cross-linking in adult Tgfbr1/2^iSMCKO^ mice exacerbates the vulnerable aorta phenotype.

**Figure 9:**
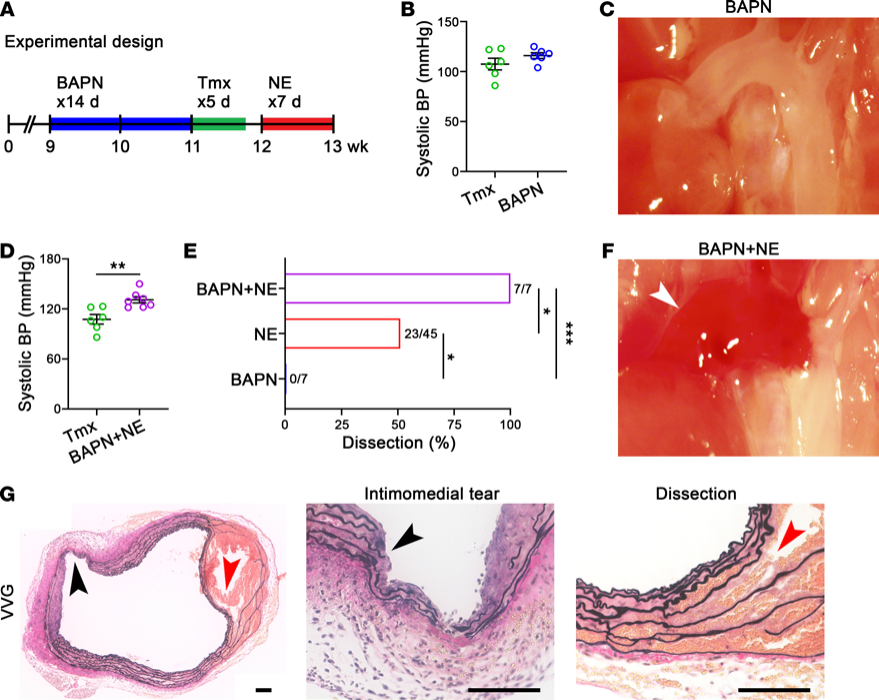
Impaired ECM cross-linking in adult mice predisposes to TGF-β–dependent aortic dissection. (**A**) Nine-week-old Tgfbr1/2^iSMCKO^ mice were given BAPN at 150 mg/kg/d for 14 days, then tamoxifen (Tmx) for 5 days, without or with NE infusion at 3.88 μg/kg/min for 7 days, and then examined at 12 or 13 weeks of age, respectively. (**B**) Systolic blood pressure (BP) measured by tail-cuff in Tgfbr1/2^iSMCKO^ mice given BAPN or not (*n* = 6). (**C**) In situ examination of BAPN-exposed Tgfbr1/2^iSMCKO^ mouse showing thoracic aorta with unremarkable appearance. (**D**) Tail-cuff blood pressure in Tgfbr1/2^iSMCKO^ mice infused with BAPN + NE or not (*n* = 6–7). (**E**) Incidence of aortic dissection in Tgfbr1/2^iSMCKO^ mice infused with BAPN (0%, *n* = 7), NE (51%, *n* = 45), and BAPN + NE (100%, *n* = 7). (**F**) In situ examination of BAPN + NE–infused Tgfbr1/2^iSMCKO^ mouse showing large mural hematoma of ascending aorta and arch (white arrow). (**G**) Verhoeff–Van Gieson (VVG) stain confirming extensive intimomedial tear (black arrows) and dissection (red arrows) of ascending aorta. Scale bars: 100 μm. Data are shown as individual values with mean ± SEM. *P < 0.05; **P < 0.01; ***P < 0.001 by unpaired Student’s *t* test (**B** and **D**) or Fisher’s exact test between infusions (**E**).

To determine whether decreased deposition of functional ECM contributes to a vulnerable aorta phenotype, we administered β-aminopropionitrile (BAPN), an inhibitor of cross-linking of newly synthesized collagen and elastic fibers, at 150 mg/kg/d s.c. for 14 days via continuous infusion. This dose of BAPN does not induce overt aortopathy in adult B6 WT mice when administered from 9 to 11 weeks of age (25) but can impair ECM integrity due to constituents that turnover rapidly, thus enabling another gain-of-dysfunction test for the dissection phenotype. We used the 2-week schedule previously reported, exposing animals to the toxin prior to TGF-β signaling disruption, as decreasing ECM synthesis would impact the efficacy of BAPN (Figure 9A). Short-term BAPN administration did not affect blood pressure or induce hemorrhagic lesions in adult Tgfbr1/2^iSMCKO^ mice (Figure 9, B and C). Histology was largely unremarkable, although with a few focal breaks of elastic fibers similar to aortas from adult B6 WT mice exposed to BAPN (Supplemental Figure 11). In contrast, BAPN exposure for 14 days followed, a week later, by NE infusion for 7 days resulted in hypertension in Tgfbr1/2^iSMCKO^ mice (as did NE alone; cf. Figure 2B) with complete penetrance of the dissection phenotype, thus exceeding disease incidence with NE alone (Figure 9, D and E). Hemorrhagic lesions were larger on gross examination, with more extensive intimomedial tears and dissection by histology (Figure 9, F and G). The marked deterioration in medial resilience after a subclinical dose of BAPN indicates a critical role for ECM integrity in TGF-β–dependent aortopathy.

### Adhesion of TGF-β receptor–deficient SMCs to defective ECM is impaired.

**Figure 10:**
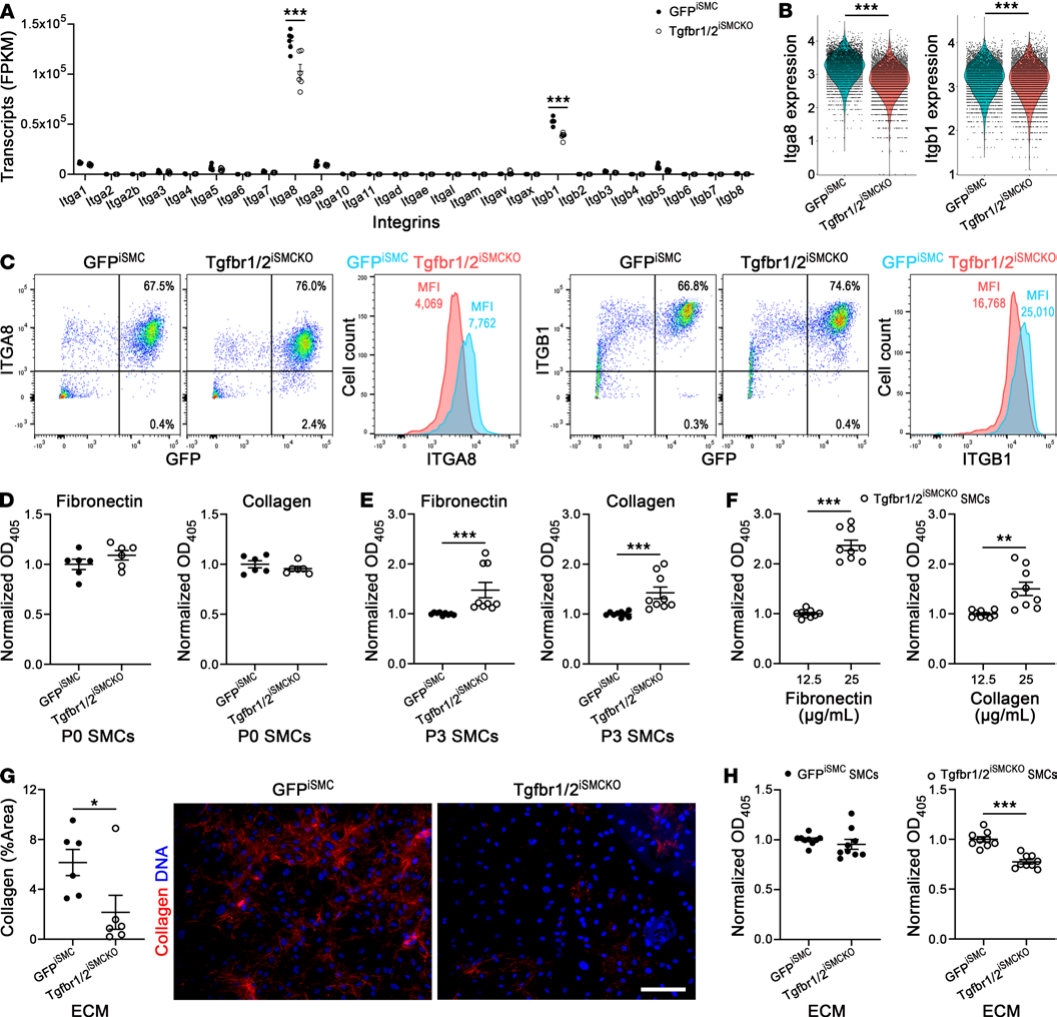
Impaired adhesion of aortic SMCs with disrupted TGF-β signaling to collagen-deficient ECM in vitro. SMCs were isolated from thoracic aortas of untreated, 12-week-old GFP^iSMC^ (closed symbols) and Tgfbr1/2^iSMCKO^ (open symbols) mice and either immediately analyzed or cultured for varying times to assess integrin expression and matrix adhesion. (**A**) Bulk RNA-seq of isolated GFP^+^ SMCs for 26 genes encoding integrins as fragments per kilobase million (FPKM) (*n* = 6). (**B**) Single-cell RNA-seq of isolated GFP^+^ SMCs for *Itga8* and *Itgb1* as corrected counts per cell (*n* = 2). (**C**) Flow cytometric analysis of isolated aortic cells for integrin α8 (ITGA8) and integrin β1 (ITGB1) cell surface expression by GFP^+^ SMCs as dot plots for percentage positive cells and histograms for MFI. (**D**) Colorimetric assay for passage 0 (P0, cultured for 3 days) SMCs adherent after 1 hour of plating to purified fibronectin- or collagen-coated plates; OD_405_ normalized to controls (*n* = 6). (**E**) Similar adhesion assay for passage 3 (P3) GFP^+^ SMCs (*n* = 9). (**F**) Adhesion assay of P3 GFP^+^ SMCs from Tgfbr1/2^iSMCKO^ mice to plates coated with different concentrations of fibronectin or collagen (*n* = 9). (**G**) Quantification by confocal microscopy of CNA35-labeled collagen (red) and DAPI-labeled nuclei (blue) in ECM elaborated by P3 GFP^+^ SMCs from GFP^iSMC^ and Tgfbr1/2^iSMCKO^ mice. Scale bar: 200 μm (*n* = 6). (**H**) Adhesion assay of P3 GFP^+^ SMCs to decellularized ECM produced by P3 GFP^+^ SMCs from GFP^iSMC^ and Tgfbr1/2^iSMCKO^ mice (*n* = 9). Data are shown as individual values with mean ± SEM. *P < 0.05; **P < 0.01; ***P < 0.001 by 2‑way ANOVA with Šidák’s multiple-comparison test (**A**), Mann-Whitney *U* test (**B**, **E**, and **G**), or unpaired Student’s *t* test (**D**, **F**, and **H**).

Because particular ECM defects were implicated as pathogenic in high blood pressure–induced aortic dissection, we investigated cell-matrix interactions in vitro and focused on integrins, transmembrane receptors linking the cellular cytoskeleton to components of the ECM. RNA-seq showed that transcripts for major SMC integrins, *Itga8* and *Itgb1*, decreased at 7 days after TGF-β signaling disruption (Figure 10, A and B). Flow cytometric analysis verified decreased MFI, but not percentage positive cells, for integrin α8 and β1 surface expression in freshly isolated aortic SMCs of Tgfbr1/2^iSMCKO^ mice (Figure 10C). Integrin α8 markedly decreased by 7–14 days of cell culture, however, corresponding to passage 1–3 SMCs, respectively (Supplemental Figure 12A). Hence, cell-matrix adhesion was assessed for nonpassaged in addition to conventional early passaged SMCs. Adhesion of passage 0 SMCs to recombinant fibronectin or collagen was similar with or without TGF-β signaling disruption (Figure 10D). By passage 3, adhesion was greater in GFP^+^ SMCs from Tgfbr1/2^iSMCKO^ than GFP^iSMC^ mice (Figure 10E), although comparisons may be confounded by differences in cell proliferation and, consequently, SMC de-differentiation. Expansion of cell numbers through 3 passages enabled further experiments, and adhesion of TGF-β receptor–deficient SMCs depended on fibronectin and collagen concentration (Figure 10F). TGF-β receptor–sufficient SMCs also demonstrated concentration-dependent adhesion to fibronectin and collagen, although with lesser magnitude differences (Supplemental Figure 12, B and C). Importantly, mutant cells exhibited decreased adhesion to collagen-deficient, decellularized ECM elaborated by SMCs from Tgfbr1/2^iSMCKO^ mice versus control matrix from GFP^iSMC^ mice (Figure 10, G and H). Together, these findings suggest that a combination of cellular dysfunction and defective ECM after TGF-β signaling disruption sensitizes to pressure-induced aortic dissection.

## Discussion

In contrast with previous reports in mice that aortopathy presents with infusion of AngII but not NE (5, 6), we found that the thoracic aorta dissected in adult Tgfbr1/2^iSMCKO^ mice with both AngII- and NE-induced elevations in blood pressure. Notwithstanding differences in mouse models (WT or germline hypercholesterolemic versus conditional disruption of TGF-β signaling), there are 2 critical differences between AngII and NE infusion. First, chronic delivery of AngII not only elevates blood pressure, it also stimulates a strong inflammatory response (26–28) that can compromise aortic wall properties (29); indeed, NE may even suppress an inflammatory response (30, 31). Second, differential AngII type 1 receptor and α1-adrenoreceptor densities along the aorta (32–34) result in greater contractions of the thoracic aorta in response to phenylephrine (an NE analog) than to AngII. Hence, it is possible that thoracic aortic wall stress is greater in AngII- than NE-induced hypertension even at the same pressure (cf. Supplemental Figure 5 in ref. 35). In the present study, both chronic infusion and bolus delivery of AngII resulted in greater elevation of blood pressure than NE despite selecting pressure-equivalent doses based on previous work (5, 6, 20), which could have contributed to the higher rates of dissection with AngII. Although inflammatory effects appear to dominate in AngII-induced hypertension, with lesions exacerbated in cases of TGF-β neutralization (28), reduction of blood pressure with hydralazine nevertheless attenuates AngII-induced aortic inflammation, stiffening, and adventitial collagen deposition (36) and it prevented NE-induced dissection herein. Regardless of vasoactive agent, the role of pressure-elevated wall stress is undeniable; aortic damage occurs when mechanical stress exceeds material strength locally whether stress is normal and strength is compromised or stress is elevated and strength is normal, or both — which appears to be the case herein due to dissection triggered by high blood pressure following short-term loss of TGF-β signaling and associated changes in ECM.

We recognize several types of injury to the vessel wall that initiate and propagate dissection of the murine aorta: (i) intimomedial entry tear, (ii) medial extravasation of blood separating SMCs from neighboring cells and fibrillar matrix, (iii) delamination of elastic laminae (widening of interlaminar distance) from accumulation of blood with traction on SMCs via intralaminar elastic fiber attachments resulting in radial reorientation of cell bodies, and (iv) further delamination with consequent fragmentation of SMCs and fibrillar matrix. Intimomedial tears extend through the intima and partially or fully through the media with the external elastic lamina and/or adventitia, preventing transmural rupture. Notably, intimomedial tears can occur in isolation without extension of lesions to rupture or dissection along axial planes (37). RBCs infiltrate among SMCs rather than along elastic laminae, suggesting medial vulnerability between cells and fibrillar matrix but not at cell attachments to intralaminar elastic fibers. Progressively, SMCs are stretched between widened elastic laminae, changing their long axis from circumferential to radial, and then rupture with membrane and cytoskeletal fragments remaining attached to elastin. In areas of greatest delamination and RBC accumulation, the elastic laminae are often stripped clean of cell and collagen fragments. The spectrum of abnormalities differs from predominant loss of SMC contacts to elastic fibers seen in aortopathy 10 weeks after conditional disruption of *Smad3* in SMCs of 6-week-old mice (38). Differences in medial injury in models of TGF-β signaling disruption may reflect a unique susceptibility of the developing versus mature aorta or a prolonged half-life of SMC–elastic fiber adhesion structures versus a shorter half-life of other SMC-ECM connections. Further temporal studies are necessary to characterize the turnover of diverse medial ECM proteins.

Although bulk passive mechanical properties of the mature ascending aortas were not statistically different between WT controls and Tgfbr1/2^iSMKO^ mice with 1 week of receptor disruption, nearly all trends (e.g., increases in wall thickness and decreases in both axial stretch and energy storage in Tgfbr1/2^iSMKO^ mice) were nevertheless consistent with statistically significant changes in descending thoracic aortas when TGF-β signaling was similarly disrupted at 11 weeks of age but evaluated mechanically after 3 weeks (24). Together, these results suggest a relatively slow but progressive deterioration of bulk properties with loss of TGF-β signaling in the adult mouse, consistent with a turnover of matrix that is characterized by decreased synthesis or increased degradation, or both. The former interpretation is supported by RNA-seq, emphasizing that TGF-β is important not only for establishing the mechanical integrity of the developing aorta (11) but also for maintaining this integrity as part of normal homeostatic processes in maturity. Among the many genes that were downregulated, reductions in competent *Col3a1*, *Col5a2*, *Lox*, and *Bgn* are consistent with thoracic aortopathies in humans (39–41), with effects of some related pathogenic variants confirmed in mice (42–45). Moreover, *COL15A1* variants associate with disease severity in families with syndromic thoracic aortic aneurysms, suggesting a genetic modifier role (46). Thus, numerous matrix constituents have nonredundant roles for structural integrity of the aortic wall.

The role of SMC contractility in this model is complex. We previously reported decreased vasoconstrictive capacity of the aorta in our pressurized ex vivo vessel preparations consistent with decreased expression of contractile molecules several weeks after TGF-β signaling disruption (11, 23, 24). Others have reported opposing results of aortic hypercontractility (associated with endothelial dysfunction) and increased expression of contractile molecules several weeks after TGF-β signaling disruption in similar strains (12, 47, 48). Certain disparities may reflect inherent differences between our biaxial (isobaric, axially isometric) vessel and uniaxial ring tests (49), the latter of which often do not account for differences in wall thickness and cannot account for altered axial stretch. In an unbiased time-series analysis of single-cell RNA-seq of *Myh11* lineage–marked SMCs, loss of contractile phenotype was mild after 1 month, moderate at 2 months, and severe at 4 months following TGF-β signaling disruption in hypercholesterolemic mice (13). In the present study, we find minor changes in contractile molecules and contractility, besides regulatory enzymes such as *Mylk4*, within 1 week of receptor deletion, suggesting that delayed loss (or gain) in SMC contractile phenotype is not a direct consequence of TGF-β signaling alone. Mechanistic experiments to determine whether changes in SMC contractility induce aortic vulnerability are limited by confounding effects on resistance vessels, except for fasudil which did not affect blood pressure at the dose used. Pervasive effects of vasodilators on aortic and resistance arteriole SMCs (50) also confound the interpretation of previous work finding blood pressure–independent aortopathy in AngII-infused and *Nos3*-deficient mice (5, 51). Furthermore, experimental designs over days to weeks with hydralazine may be susceptible to unrecognized transient high blood pressure and we did not undertake long-term vasodilator experiments.

Whereas most attention to wall integrity has focused on roles of elastic fibers and fibrillar collagens (I, III, V), the present data suggest that additional matrix and matricellular proteins may contribute substantially to wall integrity, including collagens IV, XV, and XVIII as well as connective tissue growth factor (CTGF). Types XV and XVIII collagen are multiplexins that, among other roles, associate with basement membranes and contribute to their integrity (52). Basement membrane proteins surround SMCs, albeit not uniformly, and are readily detected by immunofluorescence microscopy compared with the inconspicuous, electron-dense layer on electron microscopy (53, 54). Impaired basement membrane constituents may facilitate SMC displacement by extravasated blood and thus compound defects in the fibrillar matrix. CTGF, a prototypical TGF-β–inducible product, directly induces ECM production and facilitates that by TGF-β (55); thus, reduction in CTGF may synergize with decreased ECM transcripts after TGF-β signaling disruption. Further studies are required to elucidate roles of diverse TGF-β–dependent ECM molecules in a vulnerable aorta phenotype and conditional deletion models can describe the kinetics of disease onset for proteins with varying turnover.

An essential role for TGF-β in the mature aorta is underappreciated and underscores the continuous turnover of ECM by vascular wall cells even under quiescent conditions after postnatal development completes by 8 weeks of age (18). Previous work reported only the absence of spontaneous aortic disease after receptor deletion in adult animals (11, 56). The findings herein do not support mutually exclusive contractile versus synthetic SMC phenotypes but rather overlapping functions, including degradative properties required for protein turnover (57, 58). The turnover of total collagen in the normal adult aortic wall, based on rodent studies with radiolabeled precursors, is slow with a half-life of 70 days that changes approximately 4-fold with a reduction in half-life to 17 days with hypertension (59). Current methods to determine synthesis and degradation rates of individual proteins utilize nonradioactive, isotype-labeled amino acids and mass spectrometry, although less soluble ECM proteins are challenging to analyze. These techniques have not yet been applied to the vasculature, but protein half-lives in murine brain cortex ranged from 6, 25, 62, and 79 days for collagen XI, I, VI, and IV, respectively — other collagens were not identified (60). Besides differential turnover of individual collagens, a pool of newly synthesized collagen has more dynamic turnover than the persistent collagen network in tendons, varying with the circadian cycle versus the lifetime of the organism, respectively (61). Although we did not assess protein degradation, the markedly decreased abundance of collagen XVIII within 7 days of TGF-β signaling disruption suggests that its normal balanced turnover is not preserved. Decreases in total collagen expression were modest (~13%) 1 week after starting tamoxifen but in accordance with a greater reduction (~30%) in the aortic media at 3 weeks after receptor deletion using similar histological analysis (24). While TGF-β is necessary for ECM production by SMCs at steady state, further studies are required to determine its role under dynamic conditions imposed by hypertensive remodeling, including probable effects on ECM degradation.

We conclude that animal models with preexistent aortic vulnerabilities are more appropriate to test the clinical risk factors for aortic dissection of poorly controlled hypertension and extreme exertion. AngII and NE are relevant vasoconstrictors, as both are elevated in neurohormonal response to exercise, resulting in increased blood pressure (62). The vulnerable aorta phenotype resulting from disruption of TGF-β signaling and sensitizing to dissection elicited by transient and sustained elevations in blood pressure provides a mechanism and experimental confirmation of recent computational modeling that hypertension exacerbates but does not initiate focal mural defects leading to aortopathy, and that continual ECM turnover, either balanced or unbalanced, is required for progressive aortic disease (63). A central inference of our findings is that focal loss of medial collagen, rather than fibrosis that characterizes medial degeneration, associates with a vulnerable aorta phenotype. Conversely, we speculate that accumulation of collagen within the media may afford some protection against dissection.

## Methods

Analytical techniques are described in the Supplemental Methods.

### Sex as a biological variable.

Thoracic aortas from male mice were analyzed as the bacterial artificial chromosome containing the *Myh11-CreER^T2^* construct inserted on the Y chromosome and female mice do not express the transgene. Therefore, sex was not considered as a biological variable. It is unknown whether our findings are relevant for female mice, although similar aortopathy after disruption of TGF-β signaling was noted in females with the same construct inserted in the X chromosome (7, 64) and in male and female mice using an alternative, less specific *Acta2-CreER^T2^* construct (12).

### Mice.

C57BL/6J mice (stock 000664) and *mT/mG* mice (stock 007676) were purchased from the Jackson Laboratory, *Myh11-CreER^T2^* mice were obtained from Stefan Offermanns (University of Heidelberg, Heidelberg, Germany), *Tgfbr1^fl/fl^* mice were obtained from Martin M. Matzuk (Baylor College of Medicine, Houston, Texas, United States), and *Tgfbr2^fl/fl^* mice were obtained from Harold L. Moses (Vanderbilt University, Nashville, Tennessee, United States). Compound mutant strains were bred to a B6 background for more than 6 generations (13, 24).

### Statistics.

Graphs of quantitative data are presented as dot plots of individual values with overlying line and error bars representing the mean and SEM, respectively; single numerical values are represented by columns. Data normality was tested by Shapiro-Wilk test. Comparison of continuous variables between 2 groups with normal distribution of data was by unpaired Student’s *t* test, between 2 groups with non-normal distribution of data was by Mann-Whitney *U* test, among more than 2 groups with normal distribution of data by 1-way ANOVA (and 1‑way repeated-measures ANOVA if the same subjects were in each group) with Tukey’s multiple-comparison test for 1 independent variable, or 2-way ANOVA with Šidák’s multiple-comparison test for 2 independent variables, among more than 2 groups with non-normal distribution of data by Kruskal-Wallis test with Dunn’s multiple-comparison test, and of categorical variables between 2 groups by Fisher’s exact test. Probability values were 2-tailed and a *P* value of less than 0.05 was considered to indicate statistical significance. Bulk RNA-seq analyses were performed using DESeq2 (https://github.com/thelovelab/DESeq2); an adjusted *P* value of less than 0.05 was considered significant and fold change of greater than 2 or less than –2 was used as cutoff for differential expression. Single-cell RNA-seq analyses were performed using R (https://www.r-project.org/); markers were ranked by adjusted *P* value or by fold change if *P* values could not be differentiated.

### Study approval.

Research protocols were approved by the IACUC of Yale University.

### Data availability.

All data are included in the main manuscript and supplemental materials. Values for all data points in graphs are reported in the online Supporting Data Values file. RNA-seq data have been deposited in the NCBI Gene Expression Omnibus (GEO GSE194085). There are no restrictions on data availability.

## Author contributions

BJ, PR, CH, and GT designed the study. BJ, PR, CH, MW, SIM, MJR-R, YC, GL, and LQ conducted experiments and acquired data. BJ, PR, CH, MW, SIM, ABR, LQ, JDH, and GT analyzed and interpreted data. JDH and GT supervised the work. BJ, PR, RA, MAS, JDH, and GT wrote and edited the manuscript. The order of the 3 first authors was determined by relative effort: CH initiated the work, BJ continued the work, and PR completed the work, with all 3 collectively performing the bulk of the work and making critical contributions.

## Supplementary Material

Supplemental data

Unedited blot and gel images

Supporting data values

## Version 1 02/11/2025

In-Press Preview

## Version 2 03/24/2025

Electronic publication
