# Supplementary material for: Short-term disruption of TGF-β signaling in adult mice renders the aorta vulnerable to hypertension-induced dissection
Source: JCI Insight. 2025 Feb 11;10(6):e182629. doi: 10.1172/jci.insight.182629 (PMC11949005; doi:10.1172/jci.insight.182629)

Unedited Blots

Western blot PVDF membranes are cut according to estimated target protein size and standard molecular weight markers such that multiple different proteins of different sizes can be probed after single gel electrophoresis and membrane transfer using the limited protein extracted from single aortas.

Each membrane segment is probed with different primary antibodies and labeled with appropriate HRP-conjugated secondary antibodies, multiple membrane segments are incubated with enhanced chemiluminescence substrate, laid next to each other on a film cassette, and serial exposures are obtained at different times. Each complete or cut segment of film with different exposure are digitally scanned.

Optimal contrast images are selected, proteins with relatively weak signal have increased background signal. Image scans of composite membrane segments are shown, highlighting in red color the cropped blots shown in the manuscript.

Full unedited gel
for Figure 1B

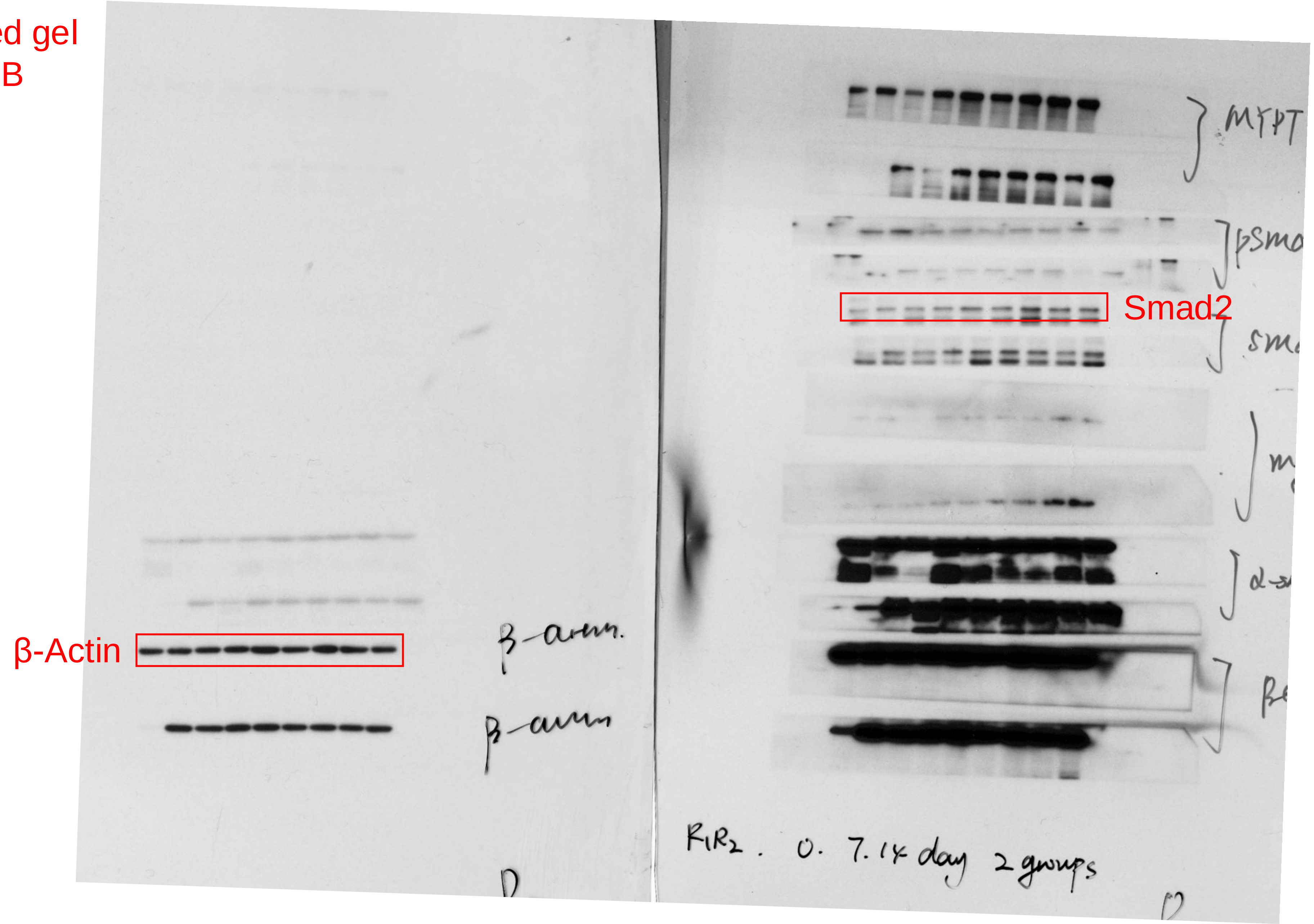

Full unedited gel for Figure 1B

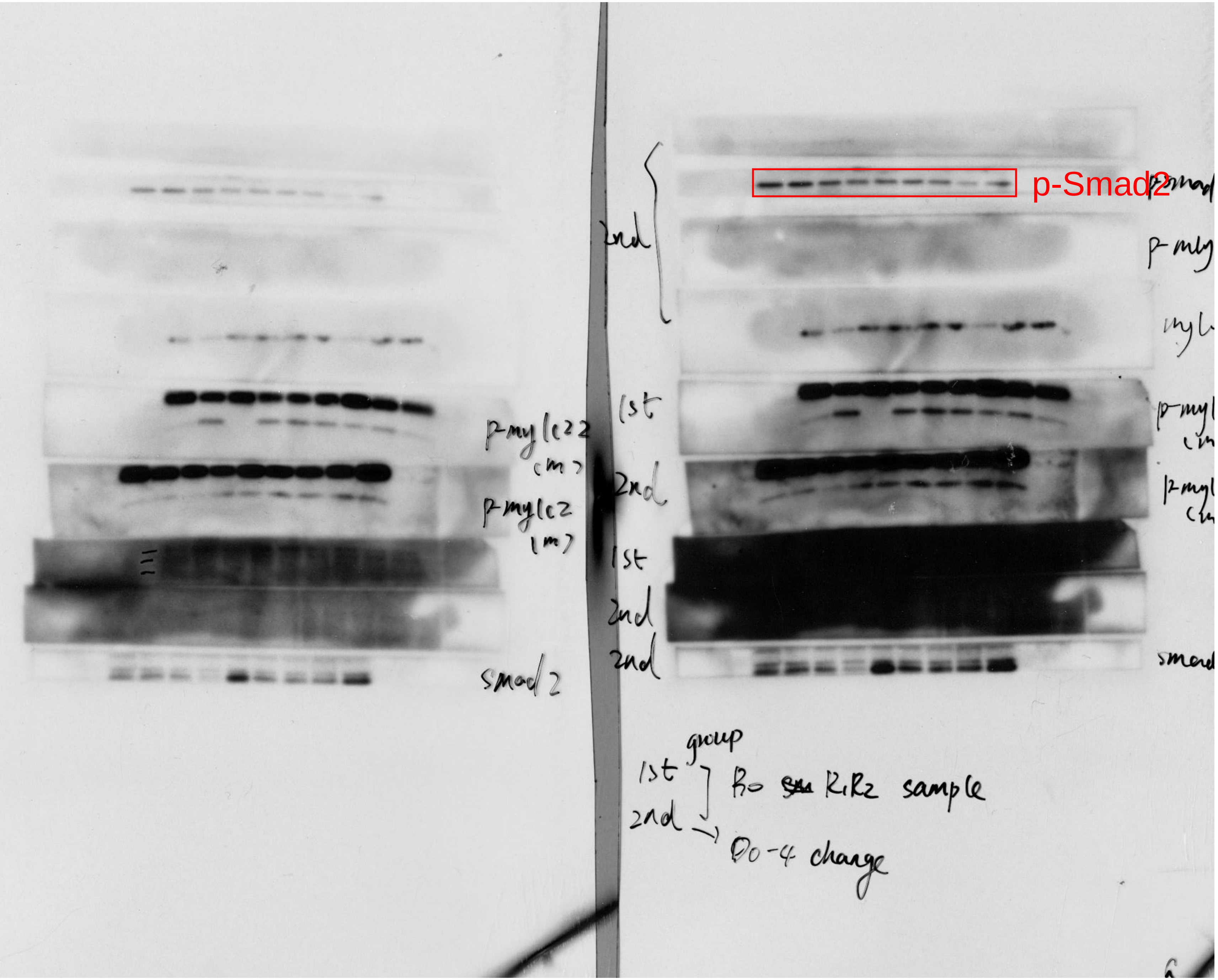

Full unedited gel
for Figure 1C

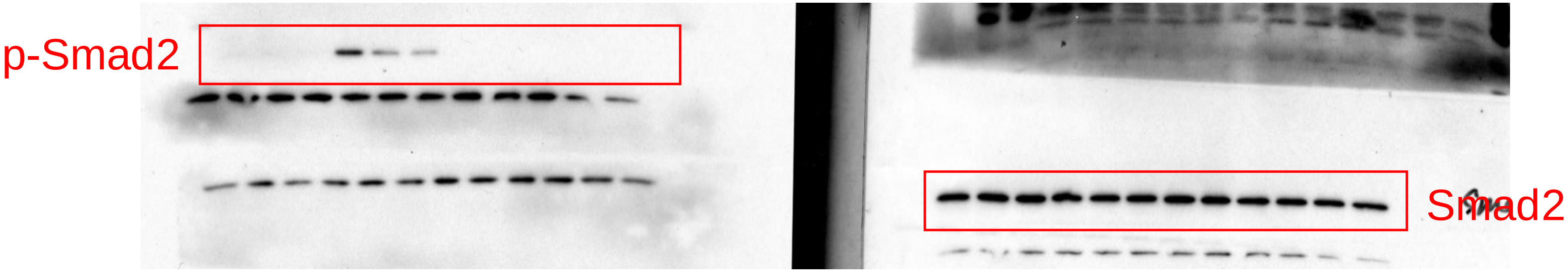

Full unedited gel
for Figure 7C

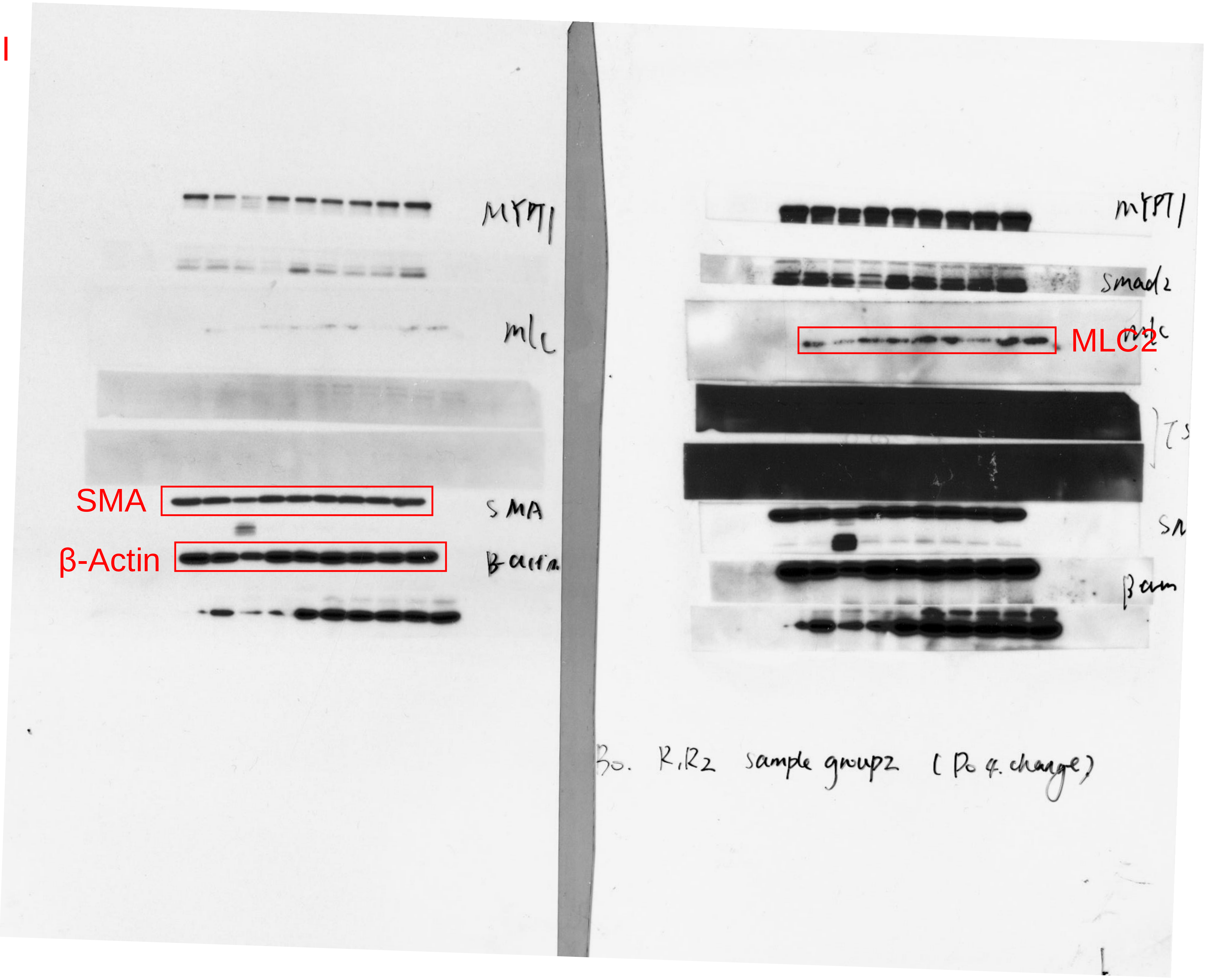

Full unedited gel for Figure 7C

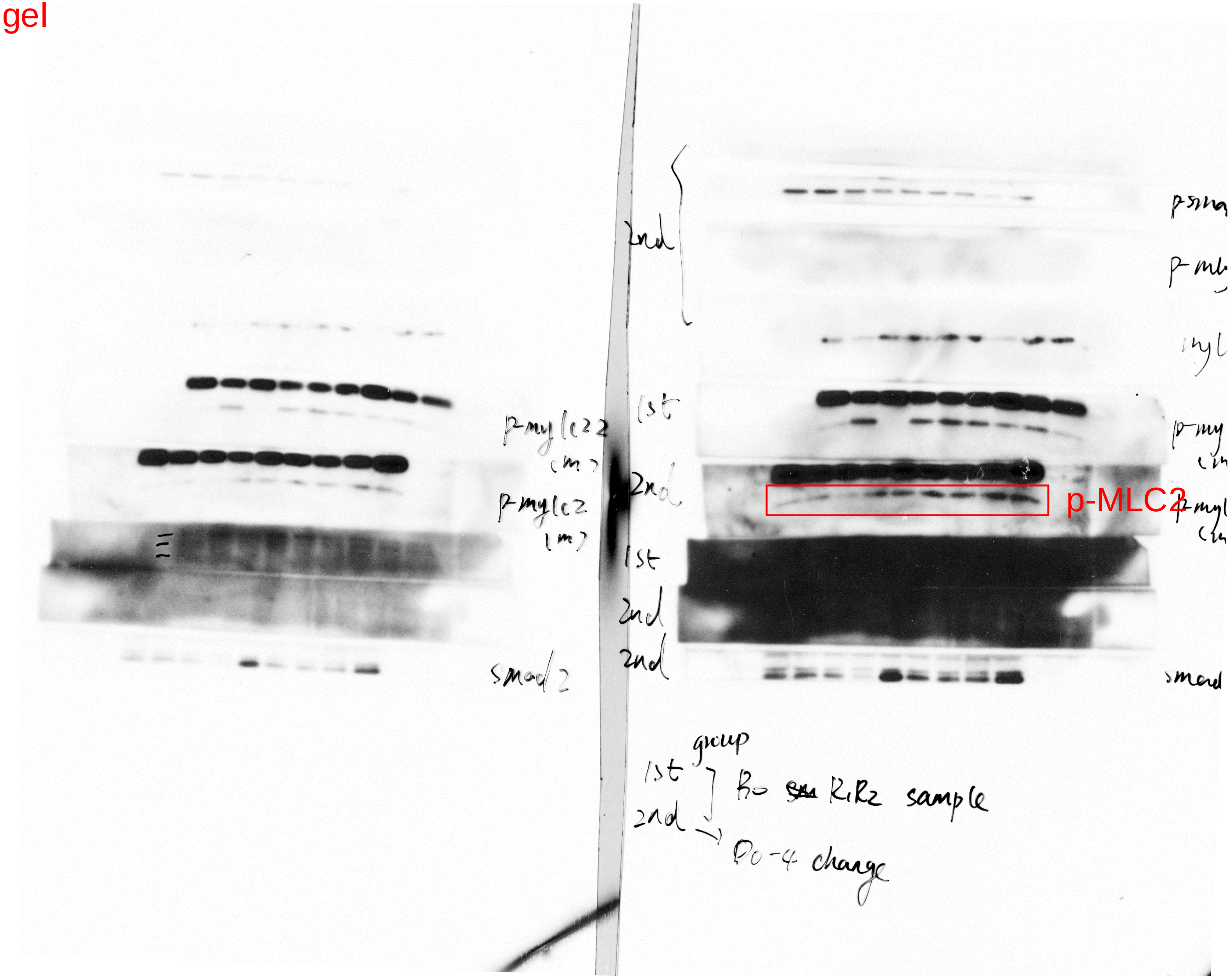

Full unedited gel for Figure 8G

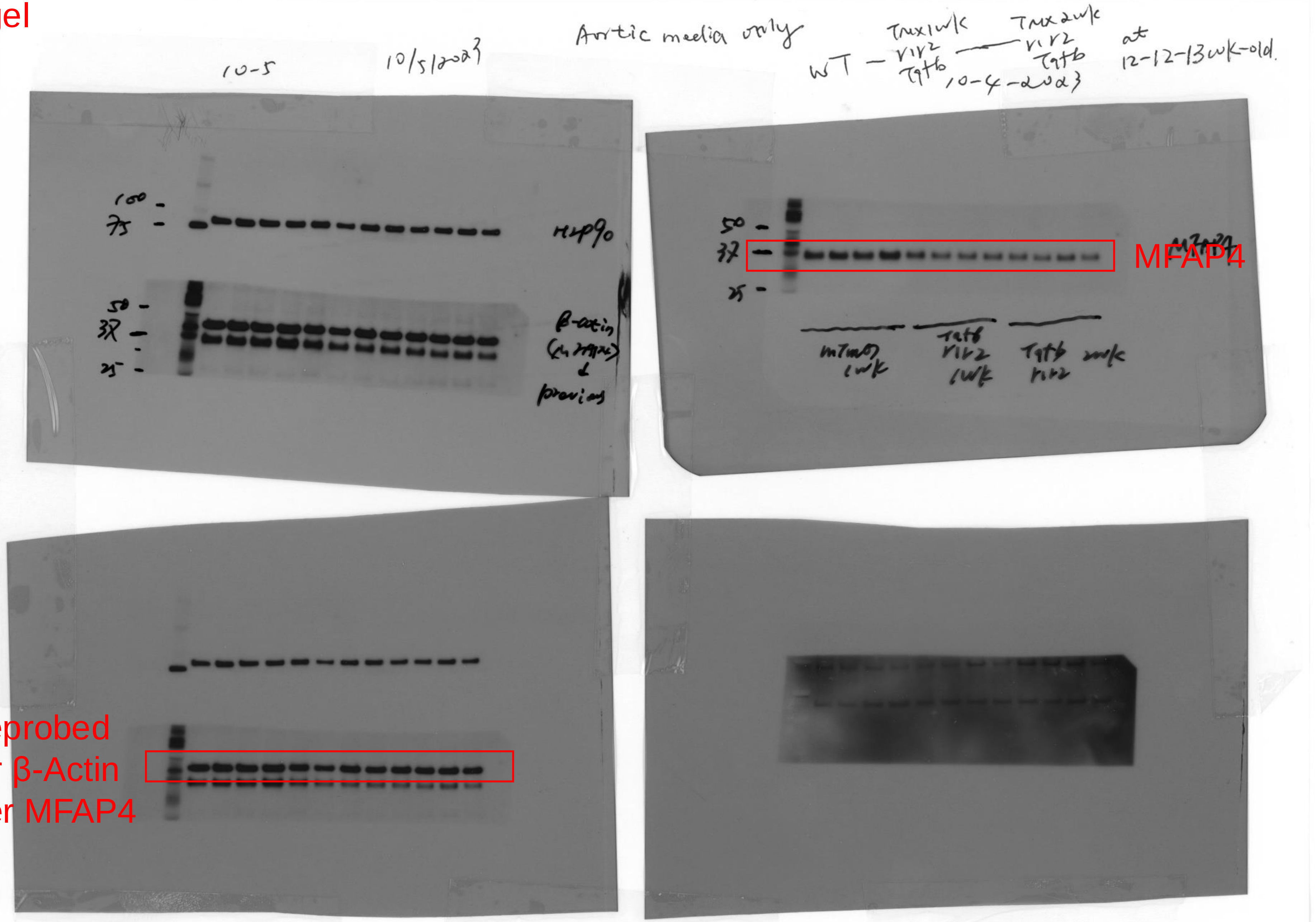

Full unedited gel
for Supplemental Figure 1A

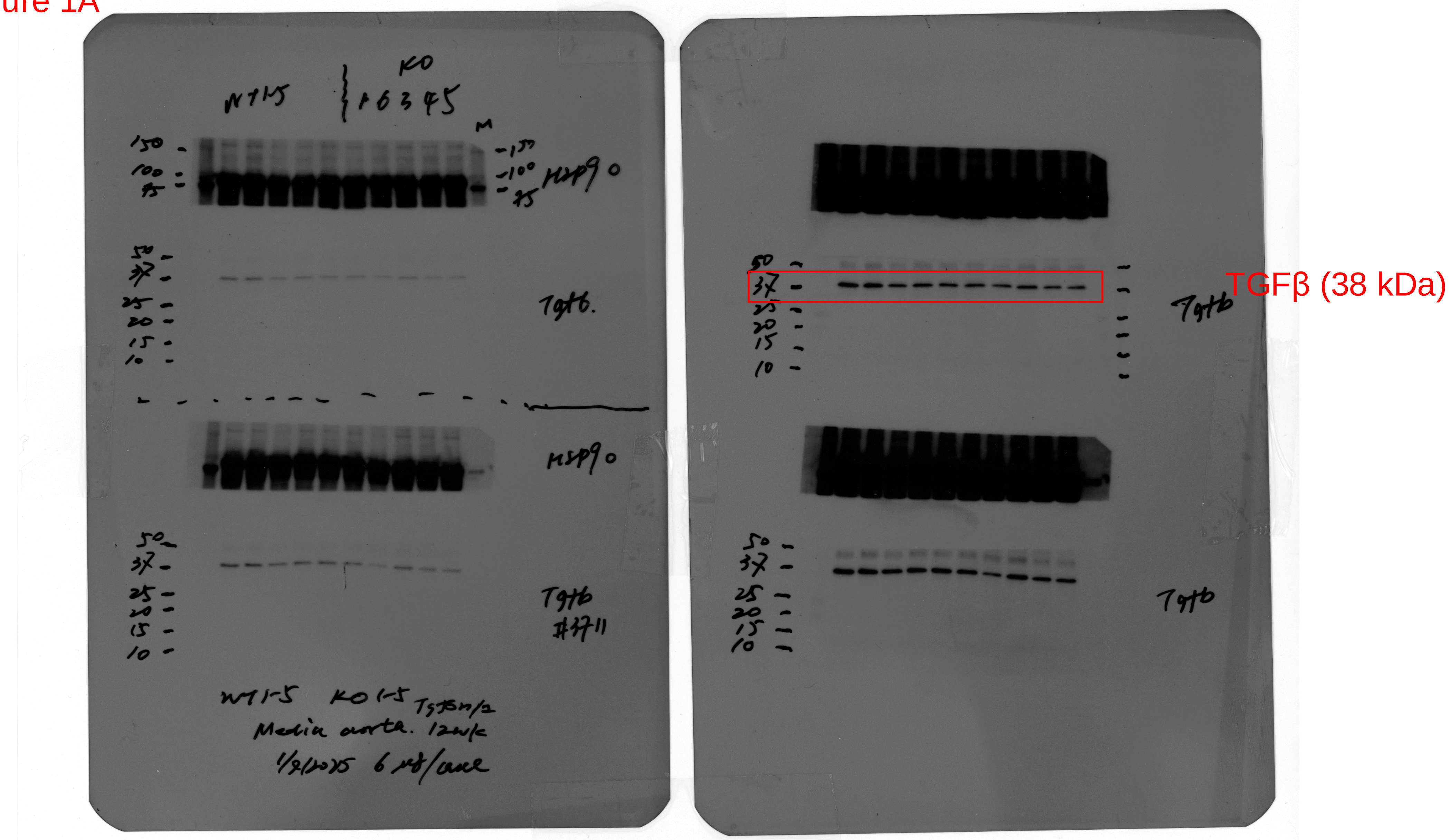

Full unedited gel
for Supplemental Figure 1A

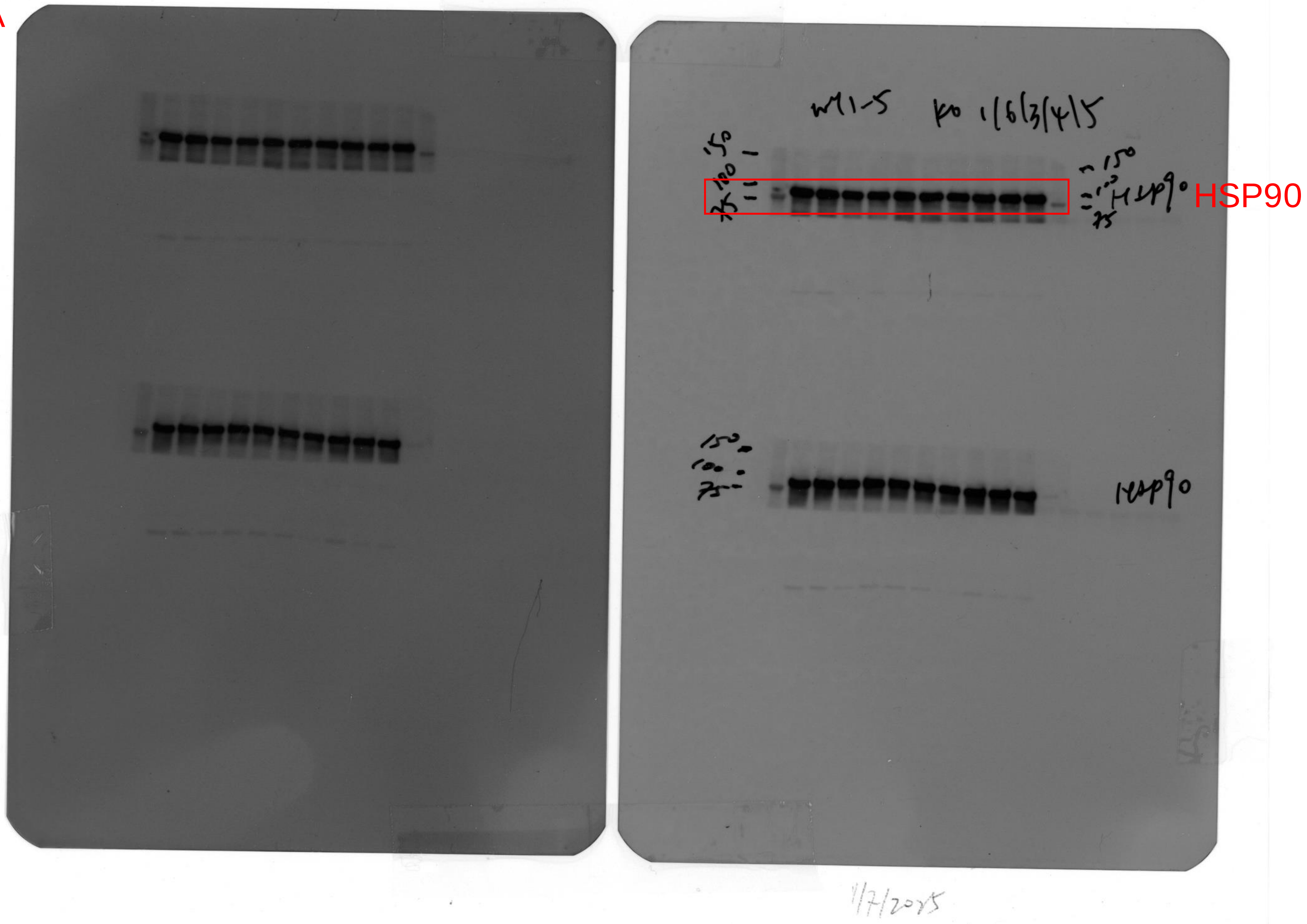

Full unedited gel
for Supplemental Figure 1B

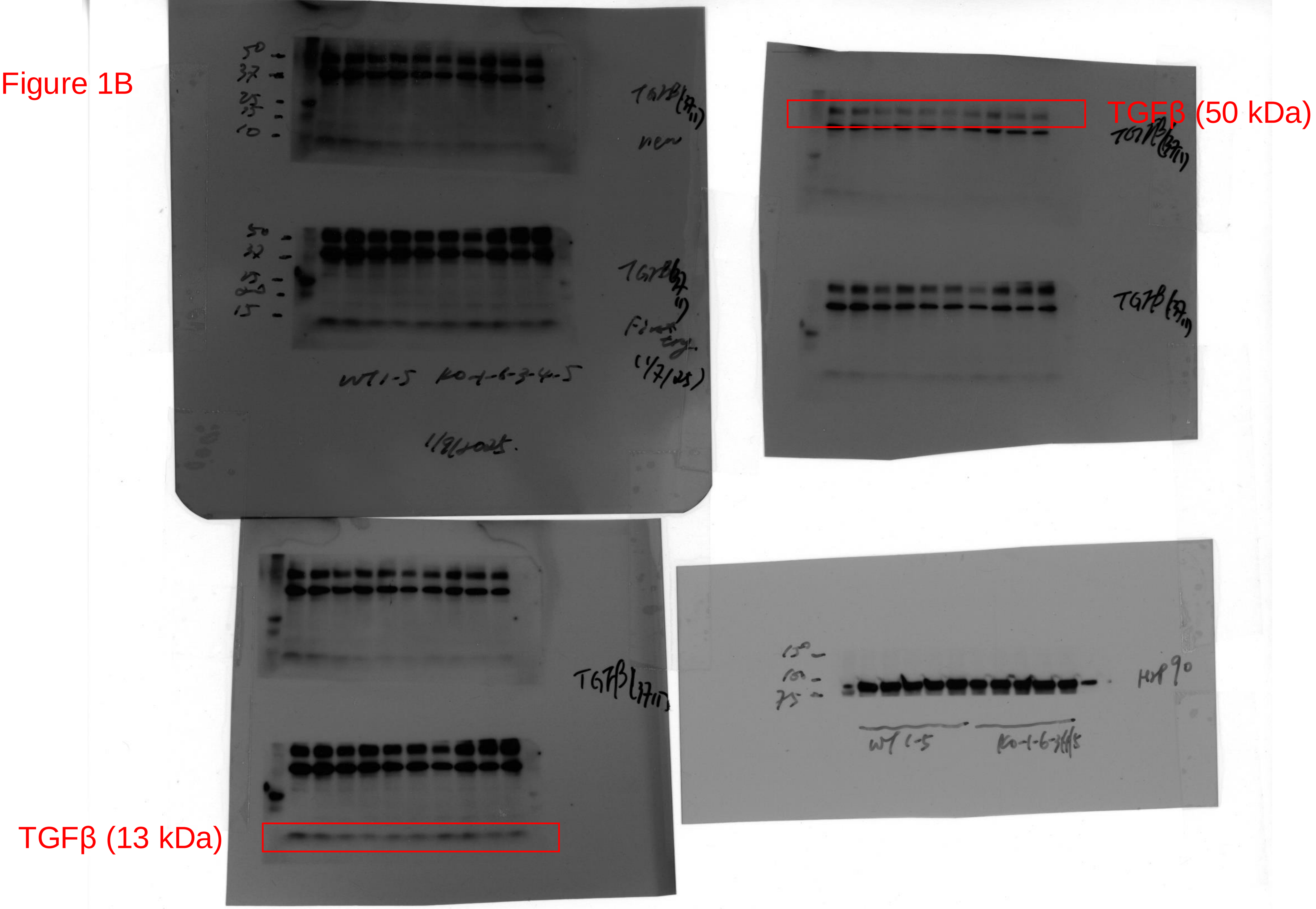

Supplement: Unedited blot and gel images [file jciinsight-10-182629-s094.pdf]
